# RhlR quorum-sensing receptor ligand sensitivity regulates the differential expression of phenazine genes in *Pseudomonas aeruginosa*

**DOI:** 10.1128/jb.00013-26

**Published:** 2026-04-08

**Authors:** Autumn N. Pope, Varun R. Bavda, Megan L. Schumacher, Alicia G. Mendoza, Anne M. Stringer, Caleb P. Mallery, Anna Czachor, Amanda F. Kurtz, Biqing Liang, Xia Ke, Joseph T. Wade, Jon E. Paczkowski

**Affiliations:** 1 Division of Genetics, Wadsworth Center, New York State Department of Health 1094, Albany, New York, USA; 2 Department of Biomedical Sciences, College of Integrated Health Sciences, University at Albany 1084, Albany, New York, USA; 3 Center for Biotechnology and Interdisciplinary Studies, Rensselaer Polytechnic Institute 8024, Troy, New York, USA; 4 The RNA Institute, College of Arts and Sciences, University at Albany 171438, Albany, New York, USA; University of Illinois Chicago, Chicago, Illinois, USA

**Keywords:** quorum sensing, gene regulation, phenazine regulation, acyl-homoserine lactone, signal transduction

## Abstract

Quorum sensing enables bacteria to coordinate gene expression in response to population density through the detection of small-molecule signals. In gram-negative bacteria, LuxR-type transcription factors bind acyl-homoserine lactones to regulate collective behaviors, yet how ligand sensitivity is tuned to shape transcriptional outcomes remains poorly defined. In *Pseudomonas aeruginosa*, the quorum-sensing receptor RhlR responds to *N*-butyryl-L-homoserine lactone (C_4_HSL) and controls late-stage quorum-sensing behaviors, including phenazine biosynthesis. Here, we use a chemical-genetic and structure-guided mutational approach to define how RhlR ligand sensitivity regulates promoter-specific transcription. We identify substitutions within the RhlR ligand-binding pocket that enhance sensitivity to C_4_HSL without altering ligand specificity, generating hypersensitive receptor variants. Increased ligand sensitivity selectively represses phenazine biosynthetic gene expression, reduces pyocyanin production, and alters phenazine output, while leaving other RhlR-dependent quorum-sensing traits unaffected. Transcriptomic and chromatin immunoprecipitation analyses reveal that these effects arise from reduced expression of the RhlR co-regulator PqsE, leading to decreased RhlR occupancy at phenazine gene promoters. These findings support a coincidence-detection mechanism in which ligand sensing and co-regulator availability jointly determine transcriptional output. Together, our results demonstrate that ligand sensitivity is a critical regulatory determinant that tunes quorum-sensing gene expression. This work reveals how changes in signal detection can reshape transcriptional hierarchies and metabolic outputs, providing insight into the fine control of bacterial collective behaviors and virulence-associated metabolism.

**IMPORTANCE:**

Quorum sensing is often described as an on/off regulatory switch, yet many bacterial behaviors require more nuanced control. This study shows that the sensitivity of a quorum-sensing receptor to its signal molecule is a key regulatory parameter that shapes downstream gene expression. By tuning the ligand sensitivity of the *Pseudomonas aeruginosa* quorum-sensing regulator RhlR, we demonstrate that changes in signal detection selectively reprogram quorum-sensing transcriptional outputs, particularly for phenazine biosynthesis, without broadly disrupting quorum-sensing functions. Our findings reveal how ligand sensing is integrated with accessory regulatory factors to control promoter selection and metabolic outcomes. This work highlights signal sensitivity as an important mechanism by which bacteria fine-tune collective behaviors and virulence-associated metabolism.

## INTRODUCTION

**Fig 1:**
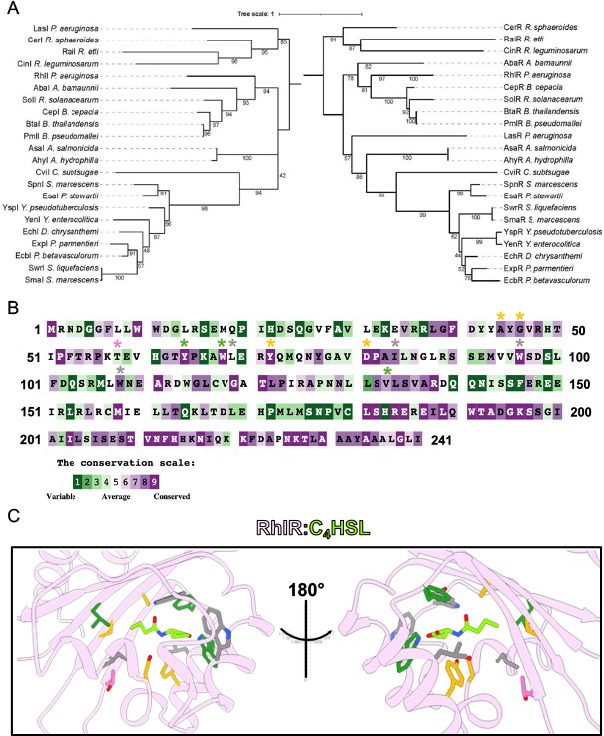
Evolutionary and structural conservation of LuxR-type transcription factor receptors. (**A**) Maximum-likelihood phylogenetic trees of RhlR and RhlI homologs. The trees were constructed based on the alignment of 22 amino acid sequences for RhlI (left) and RhlR (right) homologs from well-characterized LuxIR-type systems. Bootstrap support values, indicated at the nodes, were derived from 1,000 replicates. Species names are abbreviated, and protein names are indicated along each branch. (**B**) Conservation analysis of the RhlR protein sequence. The amino acid sequence of RhlR is colored based on conservation scores estimated using the ConsurfDB, with 300 homologs used in the analysis. Purple residues are highly conserved, and green residues are highly variable. Residues A44, G46, T58, Y64, W68, L69, Y72, D81, I84, W96, W108, and V133 are marked with asterisks and colored based on variant responses to C_4_HSL: orange = substitutions are recalcitrant to C_4_HSL; magenta = substitutions are hypersensitive to C_4_HSL; gray = substitutions are hyposensitive to C_4_HSL; dark green = substitutions were previously described and lead to a “constitutive” state for RhlR. (**C**) Zoom-in view of the RhlR (pink) LBP. C_4_HSL (green) was computationally docked into the WT RhlR LBP (PDB: 8DQ0) (27). Residues A44, G46, T58, Y64, W68, L69, Y72, D81, I84, W96, W108, and V133 are shown as sticks. Residues are colored as in panel **B**.

*Pseudomonas aeruginosa* is an opportunistic pathogen that causes thousands of deaths every year in the United States (1). Virulence and pathogenesis are largely mediated by quorum sensing (QS), a cell-cell communication mechanism that relies on the production, detection, and group-wide response to signaling molecules called autoinducers (AI) (2). Typically, an AI binds to its cognate receptor to initiate changes in gene expression, often in a positive feedback manner, to alter community behavior. *P. aeruginosa* and many other gram-negative bacteria use LuxR-type receptors to detect diffusible *N*-acyl-homoserine lactone (AHL) AI (3) (Fig. 1A). LuxR-type receptors are a class of transcription factor receptors with a highly variable N-terminal ligand-binding domain and a well-conserved C-terminal helix-turn-helix DNA-binding domain. Within the class of LuxR-type receptors, there exist three different well-characterized subclasses: (i) those that require AHL to fold, dimerize, and bind DNA to act as transcriptional activators; (ii) those that can fold and dimerize in the absence of the AHL, but require free exchange of AHL to bind DNA to act as transcriptional activators; (iii) those that can fold, dimerize, and bind DNA to act as transcriptional repressors only in the absence of AHL and whose repression is relieved by binding to AHL (3–15). To date, three LuxR-type receptors have been characterized in *P. aeruginosa*: LasR, RhlR, and QscR (7, 16–18). LasR and RhlR bind the AHLs *N*-(3-oxododecanoyl)-L-homoserine lactone (3OC_12_HSL) and *N*-butyryl-L-homoserine lactone (C_4_HSL), respectively (6, 17). 3OC_12_HSL and C_4_HSL are synthesized by the LuxI synthases LasI and RhlI, respectively. Thus, LasR and RhlR have partner synthases that produce a dedicated AHL. In each case, the receptor upregulates the expression of the partner synthase gene, initiating a positive feedback loop signaling mechanism to enhance QS traits (19–21). Conversely, QscR is an orphan receptor (i.e., lacks a partner synthase) and binds to the LasI-produced 3OC_12_HSL as well as other AHLs to regulate its activity (22). Each of the three receptors co-regulates a shared regulon as well as its own set of genes (8, 23–26).

LuxR-type receptors rely on a bipartite ligand-binding pocket (LBP) to accommodate the hydrophilic lactone head group and the hydrophobic acyl-acyl tail. Specifically, two universally conserved residues comprising a YXXXW motif are responsible for coordinating the lactone head group (Fig. 1B) (28–30). The residues responsible for binding the acyl-acyl tail are divergent among LuxR-type homologs, with the size of the hydrophobic side chains dictating the acyl chain length and the pose of the tail within the LBP (Fig. 1B). For example, LasR A127 is responsible for accommodating the C_12_ acyl tail group, one of the longer acyl chain groups known to be naturally synthesized by LuxI synthases. Substitution of A127 to a tryptophan resulted in a complete loss of activation by 3OC_12_HSL but improved the ability of the LasR A127W variant to respond to the non-cognate ligand 3OC_10_HSL compared to WT LasR (29). Conversely, SdiA, a LuxR-type protein from *Escherichia coli*, possesses a phenylalanine at the same site in the structure as LasR A127, and this results in 3OC_8_HSL being the ligand with the highest binding affinity (29). A chemical-genetic approach using AI analogs revealed that the LasR-binding site is highly malleable, allowing for multiple conformations within the LBP with different molecule orientations driving different efficacies in WT LasR (28). Specifically, residue T75 in LasR can contact the lactone head group of certain ligands to lock them in the LBP, leading to a change in conformation of the tail group, which resulted in a molecule with low efficacy. Conversely, a LasR T75V variant reversed the binding modality of the low-affinity ligand, resulting in a rotation of the lactone head group to the canonical form and a reorientation of the tail group, which coincided with increased efficacy (28).

A similar analysis of the RhlR LBP revealed that the receptor uses a distinct set of mechanisms to select ligands (10). Indicative of this, RhlR binds to C_4_- and C_6_HSL with low affinity; the C_4_HSL-RhlR interaction has an EC_50_ value of ~5 µM compared to an EC_50_ value of ~2 nM for 3OC_12_HSL-LasR, indicating that the different LBPs select ligands via different biochemical constraints (31). Indeed, when RhlR variants were expressed that were expected to enhance ligand binding, as gleaned from LasR ligand selection mechanisms, RhlR exhibited constitutive behavior. RhlR Y64F/W68F/V133F was fully active in a ligand-independent manner, indicating an LBP distinct from other characterized members of the LuxR-type family of receptors. Subsequent biochemical analyses revealed that RhlR could be stably expressed in the presence of the synthetic agonist meta-bromo thiolactone (mBTL) (10, 11). The increased stability of the RhlR:mBTL complex is likely the result of enhanced hydrophobic contacts that are absent in the RhlR LBP when it is complexed with native AHL ligands. The purification of RhlR:mBTL was an important step in characterizing other aspects of RhlR function, facilitating the discovery that RhlR binds to the metallo-β-hydrolase PqsE, a known regulator of RhlR function, to enhance the affinity of RhlR for promoter DNA (10, 27, 32–36). Full transcriptional activation of RhlR requires ligand binding and PqsE binding in both *P. aeruginosa* and heterologous *E. coli* reporter systems (34, 35, 37).

Previous structural analyses of RhlR focused on the RhlR-PqsE interface and the role of PqsE in activating RhlR at certain promoters (27, 36). In this study, we aim to understand the mechanism of RhlR ligand selection outside of the well-characterized residues (Y64/W68/V133; Fig. 1C). We discover RhlR variants at position T58, specifically substitutions to valine, leucine, and isoleucine, that result in enhanced sensitivity to C_4_HSL. We use the hypersensitive form of RhlR to better understand RhlR promoter binding and gene transcription activation at the *phzABCDEFG1/2* operons, genes that encode the enzymes responsible for the production of the phenazine pyocyanin. Recent work from our group discovered three important characteristics of phenazine gene regulation and pyocyanin production: (i) elevated levels of C_4_HSL lead to decreased levels of pyocyanin production (38); (ii) RhlR can bind at the *phzA1* and *phzA2* promoters in the absence of C_4_HSL or PqsE, but not both (37); and (iii) RhlR requires both C_4_HSL and PqsE to activate transcription initiation from the *phzA1* and *phzA2* sites (37). Here, we show that the expression of RhlR variants with increased sensitivity to C_4_HSL results in a decrease in *phz1/2* gene expression. Interestingly, the differential gene expression from these sites resulted in the loss of pyocyanin production, but not other QS-dependent traits. We perform chromatin immunoprecipitation (ChIP)-seq to establish the C_4_HSL-RhlR-binding site hierarchy at these loci and assess the phenotypic consequences of that signaling hierarchy. In total, these data establish the molecular basis for the timely expression of RhlR-C_4_HSL-dependent genes and the role of PqsE levels in modulating this expression.

## RESULTS

### RhlR ligand-binding pocket mutational analyses reveal AHL hypersensitive variants

Previous analysis of the RhlR LBP focused on highly conserved residues that were known from other AHL-binding proteins to be important contacts for AHL molecules, particularly the lactone head group. We performed conservation analyses on 300 LuxR-type receptors to determine variable and moderately conserved residues in the LBP (Fig. 1B) and, in conjunction with our previously determined experimental structure of WT RhlR bound to the synthetic agonist mBTL, used for docking C_4_- and C_6_HSL. Based on our docking simulations, C_4_- and C_6_HSL occupy a similar position in the LBP as mBTL, a highly efficacious activator of RhlR (Fig. 1C; Fig. S1). We targeted residues A44, G46, T58, L69, Y72, D81, I84, W96, and W108 (Fig. 1B and C). These residues were selected because of their proximity to the acyl tail of C_4_HSL, as well as the differences in conservation that are observed at these sites. We used an *E. coli* luciferase reporter assay to monitor ligand-dependent activation of WT RhlR and RhlR variants. WT RhlR and RhlR variants were expressed under the control of an arabinose-inducible promoter (Fig. S2). Transcription was monitored by light production via the expression of the *luxCDABE* operon controlled by the RhlR-dependent promoter of *rhlA*. Binding of RhlR to the *rhlA* promoter is highly dependent on the C_4_HSL-RhlR interaction, based on ChIP-seq analyses performed in *P. aeruginosa* as well as previously published *E. coli* reporter systems (34, 39). After expressing WT RhlR and RhlR variants in the *E. coli* reporter system, we performed dose response assays using C_4_HSL to calculate the concentration of C_4_HSL required to activate the reporter system to 50% maximum levels (EC_50_ value; Table 1). As expected, transcription activation by WT RhlR increased with increased C_4_HSL levels, with an EC_50_ value of ~2 µM. We observed a range of responses. Figure 1C depicts the targeted residues, which are colored based on the effect of amino acid substitution. Here, we focus on the substitutions that resulted in a decrease in the EC_50_ value (i.e., enhanced reporter activation was observed; pink sticks in Fig. 1C) or that resulted in an EC_50_ that could not be calculated with the concentrations that were tested (i.e., no reporter activation was observed; orange sticks in Fig. 1C).

**TABLE 1:** EC_50_ values for RhlR and RhlR variants ± PqsE^*a*^

	C_4_		C_6_	
Variant	*pqsE*	Vector	*pqsE*	Vector
WT RhlR	5.34E-07	2.42E-06	9.24E-06	1.26E-05
RhlR (T58V)	1.48E-07	6.60E-07	1.11E-07	8.09E-07
RhlR (T58L)	9.90E-08	8.54E-07	9.08E-10	1.68E-07
RhlR (T58I)	1.20E-07	1.82E-06	5.11E-08	5.61E-07
RhlR (T58F)	1.98E-07	3.21E-06	1.03E-07	5.80E-07
RhlR (T58G)	NC	NC	NC	NC
RhlR (A44M)	NC	NC	NC	NC
RhlR (G46M)	3.27E-05	NC	–	–
RhlR (G46W)	NC	NC	–	–
RhlR (L69K)	1.32E-05	NC	–	–
RhlR (L69M)	1.72E-06	1.38E-05	–	–
RhlR (L69D)	NC	NC	–	–
RhlR (Y72A)	NC	NC	–	–
RhlR (Y72T)	NC	NC	–	–
RhlR (D81N)	NC	NC	–	–
RhlR (D81K)	NC	NC	–	–
RhlR (I84M)	3.68E-06	NC	–	–
RhlR (I84W)	5.20E-06	NC	–	–
RhlR (W96A)	NC	NC	–	–
RhlR (W108F)	NC	NC	–	–
RhlR (W108Y)	NC	NC	–	–

^*a*^ “NC” indicates values could not be calculated; “–“ indicates measurement was not performed.

**Fig 2:**
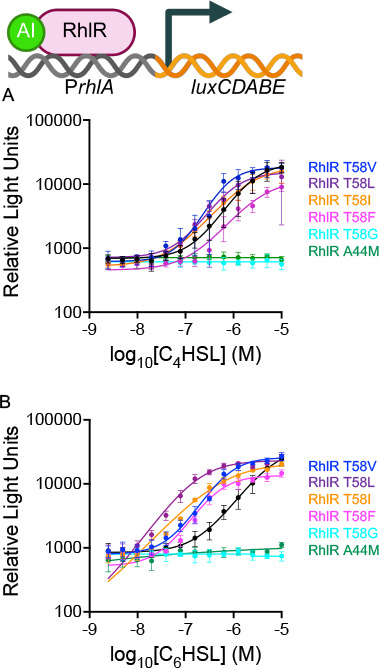
Mutational analysis of the RhlR LBP reveals AHL hypo- and hypersensitive variants. RhlR-controlled bioluminescence was measured in *E. coli*. Arabinose-inducible RhlR was expressed from one plasmid, and a p*rhlA-luxCDABE* reporter construct was carried on a second plasmid to monitor transcriptional activity. A 0.1% arabinose was used to induce RhlR. (**A**) RhlR-dependent bioluminescence was measured for WT RhlR (black) and RhlR variants (T58V = blue, T58L = purple, T58I = orange, T58F = magenta, T58G = cyan, and A44M = green) in response to increasing concentrations (µM) of C_4_HSL. (**B**) RhlR-dependent bioluminescence was measured for WT RhlR (black) and RhlR variants (T58V = blue, T58L = purple, T58I = orange, T58F = magenta, T58G = cyan, and A44M = green) in response to increasing concentrations (µM) of C_6_HSL.

The range of responses to substitutions is best highlighted by changes at residue T58 (Fig. 2A). RhlR T58 variants with substitutions to residues with the medium-sized hydrophobic side chains valine, leucine, and isoleucine resulted in decreased EC_50_ values compared to WT RhlR, indicating an enhanced binding to C_4_HSL that resulted in the increased activation of RhlR (Fig. 2A; Table 1). The RhlR T58V variant was particularly intriguing because valine occupies the same volume in the LBP but lacks the hydrophilic side chain of threonine. Thus, there is some level of hydrophobic interactions that can be tolerated at this site. Interestingly, increasing the hydrophobicity at this site by increasing the side chain length by one or two carbons (leucine and isoleucine, respectively) at T58 further decreased the EC_50_ value of RhlR (Table 1). However, there was a limit to the additive effect a hydrophobic side chain could have on RhlR activation; RhlR T58F had a slightly increased EC_50_ value (Fig. 2A; Table 1). Conversely, the loss of hydrophobicity at the site was not tolerated; a T58G substitution resulted in a complete loss in C_4_HSL activation of RhlR (Fig. 2A). Furthermore, enhancing the hydrophobicity of side chains in this particular subregion of the LBP did not generally correlate with enhanced activation by C_4_HSL, as RhlR A44M, RhlR A44W, RhlR G46W, and RhlR G46M were not activated by C_4_HSL in the reporter system, even at the highest dose of C_4_HSL that was tested (Fig. 2A; Fig. S3; Table 1). Going forward, we will use the RhlR A44M variant as a control for an RhlR variant that cannot be activated by C_4_HSL. Thus, the accessible volume of the LBP surrounding T58 appears to drive the lower efficacy of C_4_HSL activation of RhlR compared to other LuxR-type receptor/AHL pairs (29, 31, 40, 41). Indeed, of the LuxR-AHL experimentally determined structures, this region of the LBP is typically occupied by the acyl tail of long-chain AHLs or a hydrophobic residue (5, 27, 29, 30, 42, 43), a point we return to in the discussion.

### C_6_HSL ligand-dependent activation of hypersensitive RhlR variants reveals extent of hydrophobic contacts in the LBP

To determine the role of hydrophobicity in the LBP adjacent to T58, we assessed the orientation of C_6_HSL in the RhlR-binding pocket using a docking simulation (Fig. S1). We note that C_6_HSL is a naturally occurring AHL in *P. aeruginosa*, produced by the RhlI synthase that synthesizes C_4_HSL, albeit at significantly lower levels (38). It was previously shown that C_6_HSL can activate RhlR in *E. coli* reporter systems (44). Indeed, C_6_HSL activated RhlR in our reporter system, albeit with an EC_50_ value 5.5-fold higher than C_4_HSL (Fig. 2B; Table 1). Consistent with increased hydrophobic content driving receptor-ligand interactions in the RhlR LBP, C_6_HSL activated RhlR T58V/I/L variants with higher efficacy than C_4_HSL (Fig. 2B; Table 1). Interestingly, RhlR T58F had a lower EC_50_ value with C_6_HSL compared to C_4_HSL (Fig. 2B; Table 1). Together, these results indicate that the LBP of RhlR can be manipulated to enhance or reduce AHL-dependent activation. Below, we focus on RhlR residues T58 and A44 because they are representative of AHL hyper- and hyposensitive RhlR variants. Furthermore, RhlR T58 and A44 variants are biochemically tractable, making them amenable to *in vivo* and *in vitro* assessments of function.

### The PqsE-RhlR interaction enhances transcriptional activity of some RhlR variants but not others

**Fig 3:**
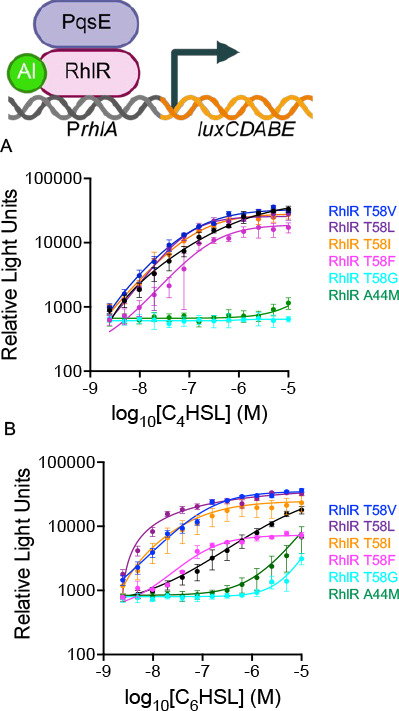
Mutational analysis of the RhlR LBP reveals AHL hypo- and hypersensitive variants in the presence of PqsE. RhlR-controlled bioluminescence was measured in *E. coli*. Arabinose-inducible RhlR was expressed from one plasmid, and a p*rhlA-luxCDABE* reporter construct was carried on a second plasmid to monitor transcriptional activity. A 0.1% arabinose was used to induce RhlR. (**A**) RhlR-dependent bioluminescence was measured for WT RhlR (black) and RhlR variants (T58V = blue, T58L = purple, T58I = orange, T58F = magenta, T58G = cyan, and A44M = green) in response to increasing concentrations (µM) of C_4_HSL and the presence of overexpressed PqsE from the pACYC plasmid. (**B**) RhlR-dependent bioluminescence was measured for WT RhlR (black) and RhlR variants (T58V = blue, T58L = purple, T58I = orange, T58F = magenta, T58G = cyan, and A44M = green) in response to increasing concentrations (µM) of C_6_HSL and the presence of overexpressed PqsE from the pACYC plasmid.

RhlR requires the accessory-binding protein, PqsE, for maximal activity. To determine if PqsE expression altered the activation of RhlR variants, we assessed AHL-dependent RhlR activation of the p*rhlA-luxCDABE* reporter in the presence of a third plasmid that constitutively expresses PqsE in *E. coli*. In this assay, PqsE enhances C_4_HSL-RhlR-dependent *rhlA* reporter expression by approximately 20-fold (Table 1), which is in agreement with findings demonstrating that PqsE can decrease the EC_50_ of the RhlR-C_4_HSL interaction at the *rhlA* promoter by up to 100-fold (39, 45). Indeed, the RhlR variants T58V/L/I that displayed enhanced activation by C_4_- and C_6_HSL (Fig. 2A and B) relative to WT RhlR could be further enhanced by the expression of PqsE (Fig. 3A and B). RhlR variants that were still active but with increased EC_50_ values for C_4_HSL could have their activity enhanced by PqsE. RhlR variants at residue L69 best exemplify this trend, as RhlR L69D/K/M initiated varying levels of transcriptional activity when treated with C_4_HSL, and all variants were similarly enhanced by the expression of PqsE (Fig. S4). Interestingly, RhlR T58F was not enhanced to the same extent by the expression of PqsE (Fig. 3A and B). Thus, alterations to the LBP can diminish the additive effect of PqsE-dependent transcription, indicating a potential role of allosteric conformational changes dependent on ligand occupancy. Additionally, the T58G and A44M RhlR variants that could not activate transcription of the reporter did not have function restored by the expression of PqsE (Fig. 2A and B). A similar trend was observed for other RhlR variants that resulted in a loss of activation by C_4_HSL; RhlR variants G46W, Y72A/T, D81K/N, and W96A (Fig. 1C; orange) were recalcitrant to C_4_HSL at all concentrations tested, and activation could not be restored by PqsE (Fig. S3 and S5). RhlR variants at residues I84 and W108 could not be activated by C_4_HSL, and thus, light levels remained low at all concentrations that were tested. Unlike the aforementioned variants, RhlR I84M/W and RhlR W108F/Y were activated by the expression of PqsE (Fig. S4). Together, these results indicate that different RhlR LBP variants are defective in activating the *rhlA* reporter, likely for different reasons, especially as it relates to activation by PqsE, a point we address with biochemical analyses.

### Purification of WT RhlR and RhlR variants bound to C_6_HSL reveals connection between PqsE and AHL binding

**Fig 4:**
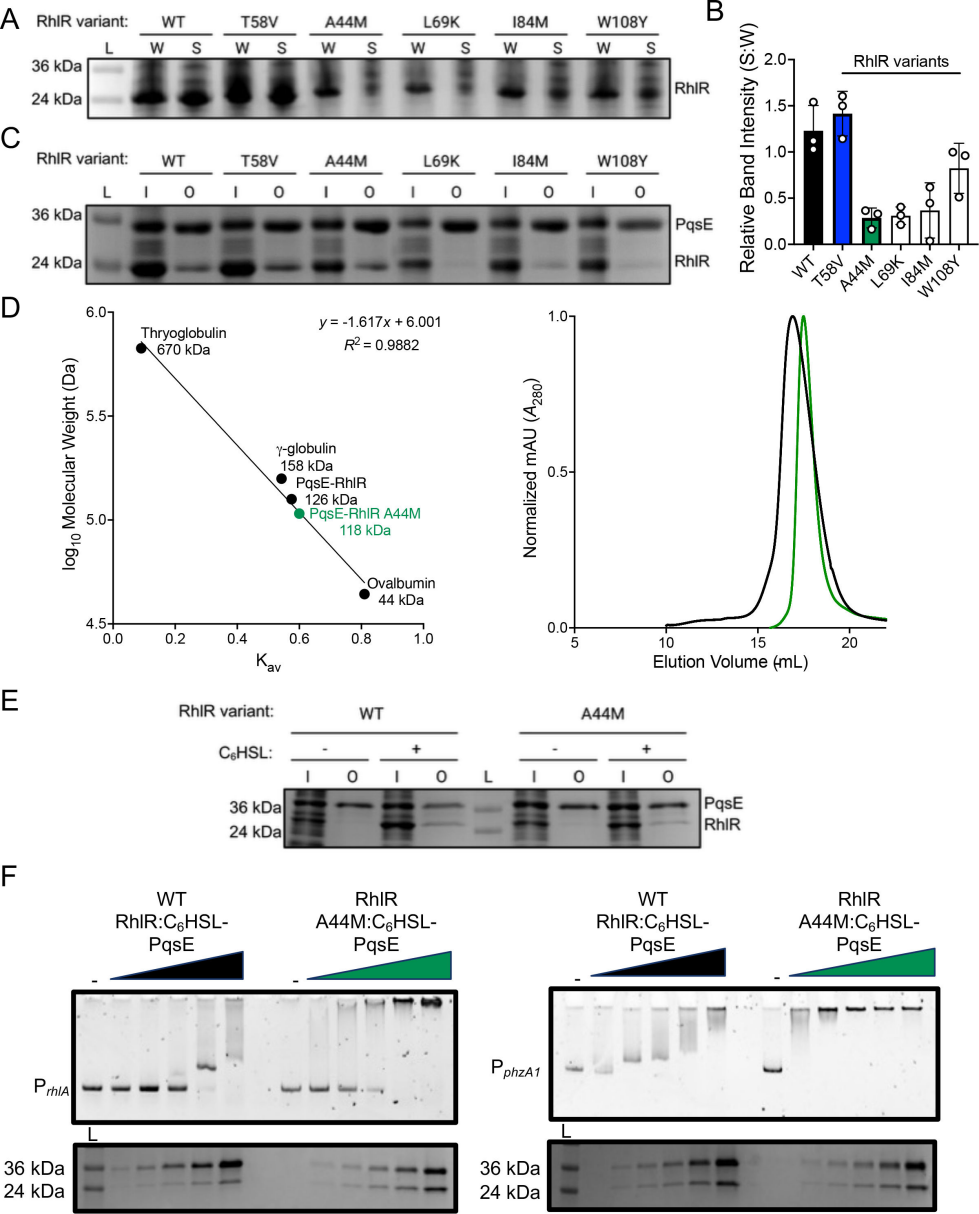
Purification and biochemical characterization of AHL hypo- and hypersensitive RhlR variants. (**A**) Total protein comparison of whole-cell lysate (W) and soluble (S) fractions from *E. coli* cells overexpressing WT RhlR and RhlR variants in the presence of 200 μM C_6_HSL during induction and lysis. “L” denotes ladder, and the 26 and 34 kDa bands are designated. (**B**) Quantification of the relative band intensities of WT RhlR and RhlR variants from three independent replicates from (**A**). Relative band intensity is a measurement of the band intensity for the soluble fraction (S) divided by the whole-cell lysate (W). (**C**) SDS-PAGE of cell lysates before (input = “I”) and after (output = “O”) affinity purification on Ni-NTA resin. Shown are WT 6x-His-PqsE-containing lysates, which were combined with lysates containing WT RhlR or RhlR variants bound by C_6_HSL. In all affinity purification experiments, RhlR does not carry a tag. “L” denotes ladder, and the 26 and 34 kDa bands are designated. (**D**) Left: size exclusion chromatography (SEC) analysis of WT RhlR (black) and RhlR A44M (green) proteins using a Superose-6 column, with protein elution volumes measured by absorbance at 280 nM (*A*_280_, *y*-axis) as a function of retention volume (mL, *x*-axis). Chromatogram traces are representative of three independent purifications for WT RhlR and RhlR A44M. Traces were normalized to a maximum value of 1 for clarity. Right Molecular weight calculations for WT RhlR (black) and RhlR A44M (green) based on molecular weight standards run using a Superose-6 column. (**E**) Same as panel **C** but no C_4_HSL was added at the time of induction. (**F**) Electrophoretic mobility shift assays (EMSA) analysis of the *rhlA* (left) or *phzA1* (right) promoter DNA alone (minus symbol, left lane) with increasing concentrations of purified WT RhlR:C_6_HSL bound to PqsE (black) or RhlR A44M:C_6_HSL bound to PqsE (green). A representative SDS-PAGE gel shows protein levels for each sample (bottom). “L” denotes ladder, and the 26 and 34 kDa bands are designated. Final concentrations of WT RhlR and RhlR A44M were: 0.094, 0.187, 0.375, 0.75, 1.5, and 3 µM per reaction.

To determine why certain inactive RhlR variants could not be fully rescued by the expression of PqsE in the *E. coli* reporter system, we assessed WT RhlR and RhlR variant solubility in the presence of AHL. Previous attempts to solubilize RhlR in the presence of C_4_HSL were unsuccessful; however, we previously showed that RhlR is soluble in the presence of C_6_HSL (46). Given that C_6_HSL can activate RhlR, we performed an RhlR solubility test on RhlR expressed in *E. coli* with C_6_HSL added at the time of induction, a key step to purifying other LuxR-type proteins such as LasR with 3OC_12_HSL. RhlR was soluble in the presence of C_6_HSL (Fig. 4A and B). We hypothesized that the RhlR variants that could not be fully rescued by PqsE were either insoluble or incapable of interacting with PqsE. To test the former, we expressed RhlR variants in the presence of excess C_6_HSL and assessed protein solubility. Many of the RhlR variants, including T58V, were as soluble as WT RhlR. The levels of RhlR variants A44M, L69K, I84M, and W108Y in the soluble fraction were significantly reduced compared to WT RhlR (Fig. 4A and B). Multiple factors may contribute to the differences in total protein in the whole-cell lysate fraction and cannot be easily disentangled. Specifically, reduced abundance of certain RhlR variants could reflect impaired expression; however, this possibility cannot be distinguished from defective ligand binding leading to increased proteolytic degradation in cells, which would also result in decreased protein levels in whole-cell fraction. If differential expression was the primary determinant of solubility, RhlR variants A44M, L69K, I84M, and W108Y would exhibit relative band intensities of 1.0 (Fig. 4B). However, quantitative analysis indicates that, among the fraction of each variant present in whole-cell lysates, solubility varied substantially, suggesting that differences in solubility are more likely attributable to binding-dependent effects rather than expression level alone. Thus, we hypothesize that, in the *E. coli* reporter system lacking PqsE, RhlR A44M, L69K, I84M, and W108Y were not binding to AHL, based on their EC_50_ values, and/or were insoluble, resulting in the loss of reporter activity (Fig. S4). We next assayed the ability of PqsE to stabilize RhlR variants in the presence of C_6_HSL. We expressed *pqsE* and *rhlR* from the pETDuet-1 vector. This more accurately simulates the *E. coli* reporter system containing *pqsE* than the results from Fig. 4A because *pqsE* and *rhlR* are co-expressed under the control of the same T7 promoter elements to ensure roughly equivalent protein levels. All RhlR variants were expressed and soluble, comparable to WT RhlR in the presence of C_6_HSL and PqsE, indicating that PqsE can have a stabilizing effect on RhlR (Fig. 4C). However, despite this stability relative to WT RhlR, PqsE could not fully rescue these RhlR variants to WT RhlR levels in the *E. coli* reporter assay (Fig. S4).

To determine if RhlR variants with substitutions in the LBP could still interact with PqsE, we used the pETDuet co-expression system to express RhlR:C_6_HSL and PqsE and then performed an affinity chromatography pulldown experiment. All of the amino acid substitutions described here are buried in the LBP and do not make direct contact with PqsE. As expected, WT RhlR and RhlR T58V interacted with PqsE at equivalent levels (Fig. 4C). Surprisingly, RhlR A44M interacted with PqsE similar to WT RhlR and PqsE (Fig. 4C). Thus, RhlR A44M was soluble and capable of binding PqsE but not being activated by AHL, indicating that binding to AHL likely induces an allosteric effect on RhlR to drive transcription. Conversely, the RhlR variants L69K and W108Y were significantly diminished in binding to PqsE, which is consistent with PqsE being incapable of fully rescuing RhlR variant activity in the *E. coli* reporter system (Fig. 4C; Fig. S4). Thus, PqsE is capable of stabilizing RhlR and enhancing its activity as a transcriptional activator in cases when an amino acid substitution in the LBP renders RhlR defective in AHL-dependent activation. Last, the RhlR variant I84M had a modest defect in PqsE binding, but this was sufficient to rescue RhlR variant activity in the *E. coli* reporter system (Fig. 4C; Fig. S4). In total, these results indicate that AHL and PqsE exert different effects on RhlR activation that can be decoupled. However, the outlier, RhlR A44M, which can bind to PqsE but not be activated by C_6_HSL, indicates that AHL binding is required to drive transcription. This is consistent with our previous ChIP-seq results that identified the *rhlA* promoter as being strongly dependent on AHL for RhlR binding and gene expression (37).

To further characterize the RhlR A44M variant, we purified WT RhlR and RhlR A44M bound to C_6_HSL. Previous purifications of WT RhlR relied on the presence of a highly efficacious AI analog to obtain soluble and stable RhlR. Indeed, this analog was required to determine the RhlR structure (27). However, we recently solubilized RhlR bound to C_6_HSL by adding AHL at the time of induction, which is standard when purifying LuxR-type receptors, as well as during cell lysis and solubilization (46). We used this approach to purify WT RhlR with high purity and yields (Fig. 4D; Fig. S6). Additionally, we purified RhlR A44M bound to PqsE (Fig. 4D; Fig. S6). Interestingly, C_6_HSL was required during induction and cell lysis to yield stable RhlR A44M bound to PqsE (Fig. 4E), indicating that RhlR A44M could bind to C_6_HSL but could not be activated by AI binding. To further characterize RhlR A44M function, we performed electrophoretic mobility shift assays (EMSA) to determine the DNA-binding capability of both WT RhlR and RhlR A44M bound to PqsE and C_6_HSL. WT RhlR:C_6_HSL-PqsE complex bound to both the *rhlA* and *phzA1* promoters (Fig. 4F), as previously described. Interestingly, RhlR A44M and PqsE induced a super shift of both the *rhlA* and *phzA1* promoters, likely reflective of enhanced binding for the Rhl box motif sequence or enhanced ability to oligomerize on the DNA. We address the ability of RhlR A44M to bind DNA in our ChIP-seq analyses below. These biochemical experiments represent two important advances to understanding RhlR structure-function: (i) WT RhlR can be purified with a “native” AI in the presence of PqsE, and (ii) a variant of RhlR incapable of being activated by AI can still be soluble in the presence of the AI and PqsE and, thus, is purifiable.

### AHL hypersensitive RhlR variants display altered phenotypic traits in *P. aeruginosa*

**Fig 5:**
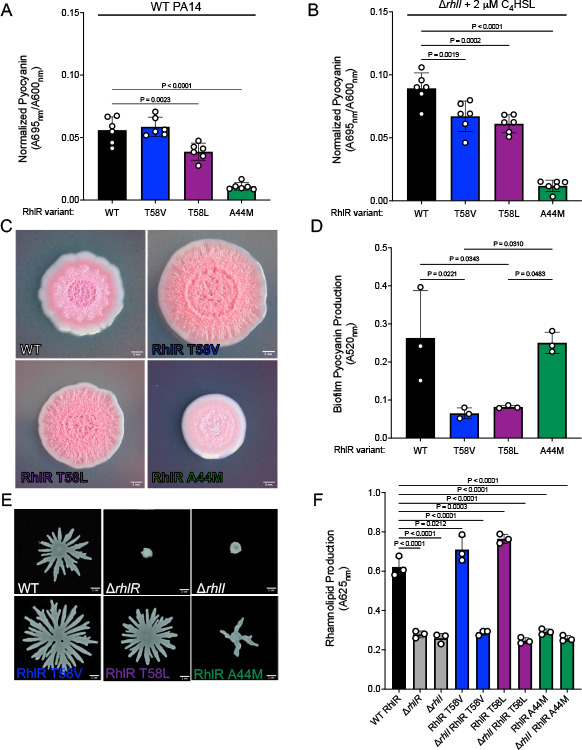
Phenotypic analysis of AHL hypo- and hypersensitive RhlR variants in *P. aeruginosa*. (**A**) Pyocyanin production was measured in WT *P. aeruginosa* strains expressing WT RhlR or the designated RhlR variants. Pyocyanin was quantified as pigment production (OD_695 nm_) divided by cell density (OD_600 nm_). (**B**) Same as panel A except pyocyanin production was measured in a *P. aeruginosa* Δ*rhlI* strain expressing WT RhlR or the designated RhlR variants in the presence of 2 μM C_4_HSL. (**C**) Representative images of Congo red colony biofilm plates for strains of *P. aeruginosa* expressing WT RhlR, RhlR T58V, RhlR T58L, and RhlR A44M. (**D**) Pyocyanin production from extractions obtained from colony biofilms grown in panel C. Extracted pyocyanin was measured at 520_nm_. (**E**) Swarming motility assay on 0.4% agar using *P. aeruginosa* strains expressing WT RhlR or the designated RhlR variants. (**F**) Rhamnolipid production of the supernatants from the designated strains grown in LB for 24 h using Victoria Pure Blue BO and measuring absorbance of the dye at OD_625 nm_. Statistical analyses for all assays were performed using an ordinary one-way analysis of variance with a Dunnett’s multiple comparisons test. All comparisons were made to the WT control strain; comparisons that were deemed not significant by these analyses are not shown.

To assess the consequences of RhlR hypersensitivity to AHL molecules, we performed phenotypic analyses of well-characterized RhlR-dependent QS traits, such as pyocyanin, biofilms, swarming, and rhamnolipid production, in strains expressing *rhlR* mutants from the native *rhlR* locus. The RhlR variant proteins were expressed to similar levels as WT RhlR (Fig. S7A). We previously showed that pyocyanin production via the regulation of the *phzABCDEFG1/2* operon by RhlR was sensitive to levels of C_4_HSL; C_4_HSL concentrations below ~500 nM and above ~5 µM resulted in a loss of pyocyanin production (38). Thus, we hypothesized that expression of a RhlR variant with higher affinity for C_4_HSL would repress pyocyanin production at high-cell density due to hyperactivation of the Rhl system. Indeed, we observed this phenomenon in strains producing their own C_4_HSL (i.e., an otherwise WT background), as strains expressing RhlR T58L but not RhlR T58V produced less pyocyanin than WT (Fig. 5A). Additionally, a strain expressing RhlR A44M was unable to produce detectable levels of pyocyanin, indicating a loss of RhlR-dependent signaling via C_4_HSL (Fig. 5A). To ensure that the increased sensitivity to C_4_HSL by the RhlR variants and not the dysregulation of *rhlI*, which would alter the C_4_HSL concentrations in these backgrounds, led to the decrease in pyocyanin production, we measured pyocyanin levels in a Δ*rhlI* strain expressing WT RhlR and RhlR variants in the presence of 2 µM C_4_HSL. Supplementation of a Δ*rhlI* strain expressing RhlR T58V or T58L with 2 µM C_4_HSL resulted in less pyocyanin produced by the culture compared to a Δ*rhlI* strain expressing WT RhlR treated with 2 µM C_4_HSL (Fig. 5B), indicating that RhlR sensitivity to C_4_HSL is important for the regulation of phenazine gene expression. Thus, pyocyanin production can be suppressed when RhlR is overactivated by AHL.

To understand if C_4_HSL sensitivity affected phenazine production in the context of biofilms, we performed analyses on the above strains to assess their ability to produce pyocyanin and form colony biofilms on Congo red plates. WT *P. aeruginosa* produced a stereotypical colony biofilm with a rugose center and smooth outer edge (Fig. 5C). WT *P. aeruginosa* strains expressing RhlR T58V and T58L variants produced hyper-rugose colony biofilms with a smaller smooth periphery (Fig. 5C). Conversely, a WT *P. aeruginosa* strain expressing the RhlR A44M variant produced markedly different colony biofilms with a largely smooth texture (Fig. 5C). The colony biofilm formed by the strain expressing RhlR A44M further confirms that RhlR signaling is intact via PqsE but not through the activation by C_4_HSL. Furthermore, it was previously shown that a Δ*pqsE* strain phenocopies the hyper-rugosity of a Δ*rhlR* strain (33). Thus, we hypothesize that the strain expressing a RhlR T58L variant is hyper-rugose because its enhanced binding to C_4_HSL has a subsequent effect on PqsE levels, which we address below. Additionally, these phenotypes are consistent with previous findings that showed that Δ*rhlI* strains exhibit a smooth colony morphology (33, 47).

Colony morphology and rugosity often correlate with phenazine production; deletion of the phenazine operons leads to a hyper-rugose colony morphology similar to that of a Δ*rhlR* strain (47). The hyper-rugose phenotype is thought to function as a mechanism to compensate for the loss of pyocyanin, which can act as a terminal electron acceptor under anoxic conditions. Thus, we hypothesized that strains expressing RhlR variants that were hypersensitive to C_4_HSL would produce less pyocyanin compared to WT or the strain expressing the RhlR A44M variant (Fig. 5D). We extracted pyocyanin from the Congo red biofilm plates and measured the absorbance at 520_nm_. Strains expressing the RhlR T58V and T58L variants produced significantly less pyocyanin than the WT strain or the strain expressing the RhlR A44M variant (Fig. 5D; Fig. S7B). Thus, while the strain expressing the A44M variant is defective in pyocyanin production in planktonic culture, it is still capable of producing pyocyanin when grown in a biofilm. This is consistent with previous findings that showed that *rhlI* mutants are smooth, express the phenazine genes, and produce pyocyanin, which is in contrast to *rhlR* mutants grown as planktonic or biofilm cultures (10, 47). Thus, the strain expressing the RhlR A44M variant can still drive gene expression under certain environmental conditions, but, given that colony morphology is not the same as the WT phenotype, it is likely context specific (i.e., only certain RhlR-dependent genes can be regulated). This context-specific pyocyanin production is also likely due to differential regulation of the *phzABCDEFG1/2* operons depending on bacterial growth state. *phz1* is more highly expressed in planktonic culture and is hypothesized to be more C_4_HSL sensitive than *phz2,* which is expressed at higher levels in biofilms (37, 48). Both operons have RhlR-dependent expression; therefore, the RhlR A44M variant may illustrate RhlR-dependent expression that does not require C_4_HSL activation. Together, this establishes that colony biofilm formation and pyocyanin production correlate with RhlR sensitivity to AHL.

To determine if sensitivity to C_4_HSL specifically repressed pyocyanin production or if it was a generalized response to suppress other QS traits, we performed additional phenotypic analyses of the above strains to assess the ability of *P. aeruginosa* to swarm on low percentage agar plates, a trait known to be dependent on the RhlR-AHL interaction (49, 50). As expected, WT *P. aeruginosa* was capable of swarming, and a Δ*rhlI* strain was not (Fig. 5E; Fig. S7C). Distinct from the observed pyocyanin production phenotypes, strains expressing RhlR T58V and T58L were capable of swarming and matched the total swarming diameter of WT *P. aeruginosa* (Fig. 5E; Fig. S7C), indicating that not all *P. aeruginosa* RhlR-dependent QS phenotypes are suppressed by hypersensitivity to AHL. Conversely, a strain expressing RhlR A44M was incapable of swarming to WT levels, consistent with the inability of the variant to be activated by AHL (Fig. 5E). We next correlated the ability to swarm with rhamnolipid production, a well-characterized C_4_HSL-RhlR-dependent trait. The ability of strains to swarm was consistent with rhamnolipid production as measured by the absorbance of Victoria Blue PO with cell-free supernatant (Fig. 5F). Furthermore, the hyperactive strains required the presence of C_4_HSL to produce rhamnolipids, as all RhlR variants in a △*rhlI* background were defective in rhamnolipid production. Thus, rhamnolipid production and the subsequent swarming of the colony correlate with RhlR sensitivity to AHL.

### Enhanced selectivity of RhlR for C_4_HSL leads to differential gene expression

**Fig 6:**
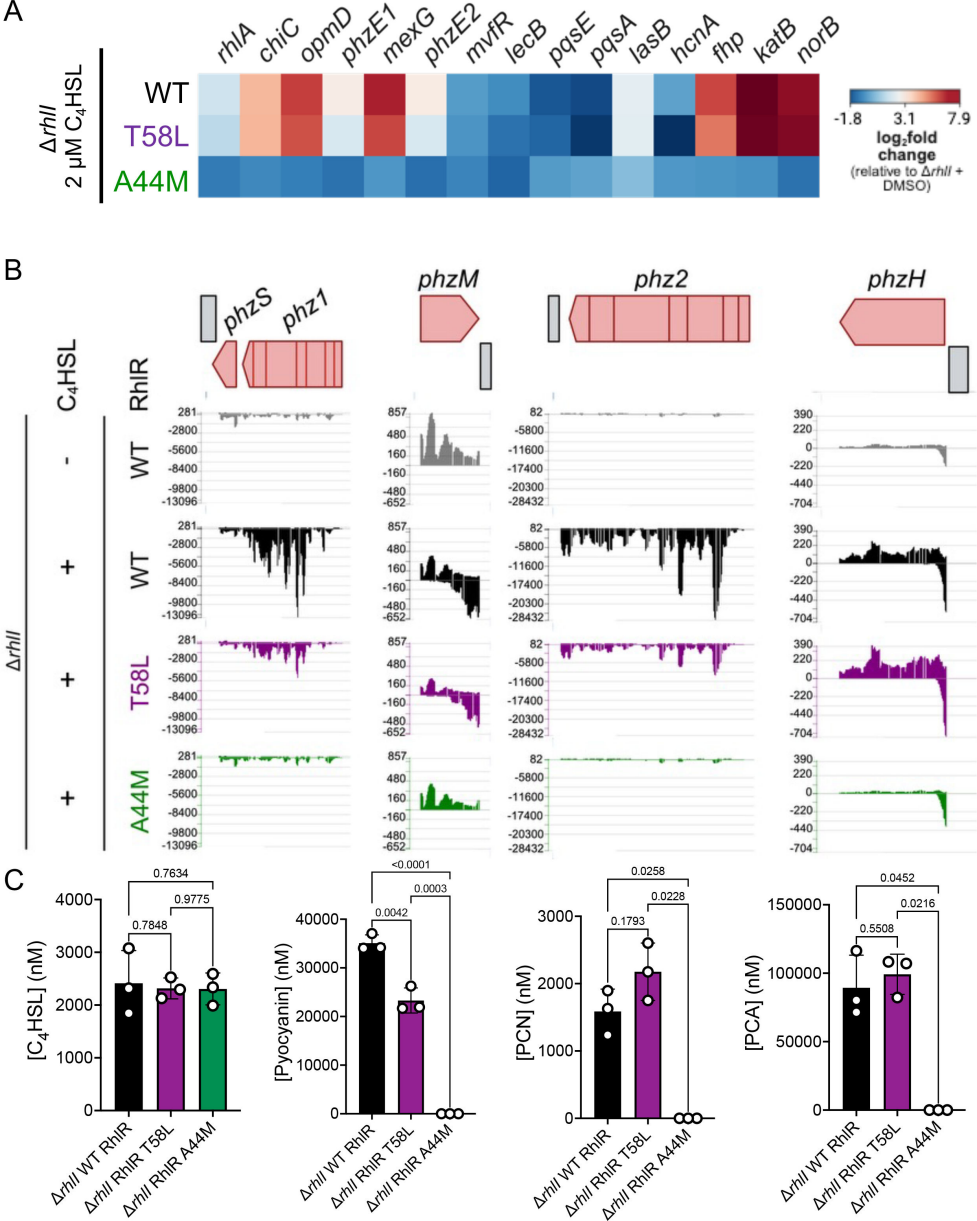
RhlR variant activation alters expression of phenazine genes and pyocyanin production. (**A**) Heat map of normalized gene expression between Δ*rhlI* strains expressing WT RhlR in the presence of 2 μM C_4_HSL with Δ*rhlI* strains expressing RhlR A44M or RhlR T58L variants in the presence of 2 μM C_4_HSL. (**B**) Signal map profiles of the designated strains showing the level of mapped RNA reads to all phenazine genes: *phzABCDEFG1/2* operons, *phzM*, *phzS*, and *phzH*. (**C**) Absolute concentrations of C_4_HSL, pyocyanin, phenazine-1-carboxylic acid (PCA), and phenazine-1-carboxamide (PCN) from cell-free supernatants of WT strains expressing WT RhlR and RhlR variants T58L and A44m using ultra-high-performance liquid chromatography (UHPLC) coupled with a high-resolution mass spectrometer. All strains were supplemented with 2 µM C_4_HSL. Bars represent the mean of three biological replicates. Statistical analyses for all compound measurements were performed using an ordinary one-way analysis of variance with a Tukey’s multiple comparisons test.

To determine the effects of hypo- and hypersensitivity to C_4_HSL on RhlR-dependent transcription activation in *P. aeruginosa*, we performed RNA-seq on Δ*rhlI* strains expressing WT RhlR, RhlR T58L, and RhlR A44M in the presence of 2 µM C_4_HSL. We chose this approach to better control the levels of C_4_HSL across samples. All gene expression analyses were done relative to a Δ*rhlI* strain without exogenous supplementation of AHL. Thus, we examined the total increase in C_4_HSL-dependent gene regulation. We observed increased *rhlA* RNA levels in strains expressing WT RhlR and the RhlR T58L variant compared to Δ*rhlI* without C_4_HSL or a strain expressing the RhlR A44M variant (Table 2; Table S1). Additionally, many other genes that were previously reported as dependent on the RhlI-synthesized AI showed similar trends; *lasB*, *mexG*, *katAB*, and *chiC* were expressed at high levels when a hypersensitive RhlR variant was expressed, similar to WT RhlR (Fig. 6A; Table 2). To corroborate our RNA-seq data, we built an *rhlA* promoter fusion to mScarlet and assayed reporter levels in the same genetic backgrounds as our transcriptomic experiment (Fig. S7D). Strains expressing hypersensitive RhlR variants produced similar levels of fluorescent signal relative to the WT strain, while a strain expressing a hyposensitive RhlR variant had diminished levels of fluorescent reporter (Fig. S7D). Furthermore, the *rhlA* transcript levels in both of our assays are consistent with the swarming and rhamnolipid production data in Fig. 5E and F. Additionally, we note that not all genes that were upregulated in a C_4_HSL-dependent manner are the result of direct regulation by RhlR, as many genes with altered expression did not have a corresponding RhlR-binding site in their promoter region, as defined by our previous ChIP-seq analyses (37).

**TABLE 2:** Relative gene expression for QS genes of interest

			Expression value				Log_2_fold change		
Name	Synonym	Product	Δ*rhlI* WT RhlR dimethyl sulfoxide	Δ*rhlI* A44M C_4_HSL	Δ*rhlI* WT C_4_HSL	Δ*rhlI* T58L C_4_HSL	Δ*rhlI* A44M C_4_HSL	Δ*rhlI* WT RhlR C_4_HSL	Δ*rhlI* T58L C_4_HSL
*norC*	PA14_06810	Nitric-oxide reductase subunit C	20,042.5	12,922.5	3,120,586.5	3,026,578.5	−0.633177272	7.282610918	7.238481486
*norB*	PA14_06830	Nitric-oxide reductase subunit B	40,736.5	28,591	6,989,112.5	7,897,306	−0.510760951	7.422643427	7.59889475
*katA*	PA14_09150	Catalase	3,102,635	3,887,333	14,093,439.5	12,151,161	0.325286713	2.183457853	1.969528274
*phzS*	PA14_09400	Hypothetical protein	748,424	303,377	726,300.5	377,065.5	−1.302744108	−0.04328925	−0.989040667
*phzG1*	PA14_09410	Pyrodoxamine 5'-phosphate oxidase	203,791.5	118,586	560,110.5	312,421.5	−0.78116018	1.458617595	0.61639986
*phzF1*	PA14_09420	Phenazine biosynthesis protein	229,001.5	137,969	1,981,300.5	1,047,752.5	−0.731012901	3.113018754	2.19386901
*phzE1*	PA14_09440	Phenazine biosynthesis protein	405,582.5	282,659.5	4,232,986.5	2,058,712.5	−0.520930219	3.383608578	2.343675061
*phzD1*	PA14_09450	Phenazine biosynthesis protein	218,118.5	153,729	2,329,509	1,058,250	−0.504722793	3.41684186	2.278496444
*phzC1*	PA14_09460	Phenazine biosynthesis protein	219,761	135,611.5	655,136.5	272,395.5	−0.696455855	1.575860148	0.30976749
*phzB1*	PA14_09470	Phenazine biosynthesis protein	337,590.5	163,479	218,602.5	73,793	−1.046168983	−0.626964406	−2.193718432
*phzA1*	PA14_09480	Phenazine biosynthesis protein	29,990	8,661	15,501	6,068	−1.791876009	−0.952120233	−2.305188532
*phzM*	PA14_09490	Phenazine-specific methyltransferase	346,737	93,038	184,602	79,302.5	−1.897949804	−0.90942361	−2.128403541
*opmD*	PA14_09500	Outer membrane protein	7,113	6,012	631,843.5	525,347	−0.242613154	6.472965291	6.206668687
*mexI*	PA14_09520	RND efflux transporter	17,503.5	15,683.5	920,093.5	830,527.5	−0.158395879	5.716065138	5.568312601
*mexH*	PA14_09530	RND efflux membrane fusion protein	9,619	11,247.5	903,148	723,774.5	0.225645544	6.552931695	6.233509551
*mexG*	PA14_09540	Hypothetical protein	4,056.5	4,729.5	516,840.5	332,571.5	0.221452182	6.993339826	6.357285242
*lasB*	PA14_16250	Elastase	3,198,853	6,614,384	19,914,902.5	19,328,123.5	1.048052108	2.638221815	2.595074977
*rhlA*	PA14_19100	Rhamnosyltransferase chain A	252,582	200,100.5	1,118,561.5	879,447.5	−0.336027058	2.146820844	1.799845626
*rhlB*	PA14_19110	Rhamnosyltransferase chain B	132,181	165,369.5	1,056,112.5	741,717	0.323178359	2.998176802	2.48835402
*rhlR*	PA14_19120	Transcriptional regulator	638,041	581,659	1,082,023	906,213	−0.133475518	0.762010128	0.506201054
*lecB*	PA14_20610	Fucose-binding lectin PAIIL	356,932	208,420	366,190	250,651	−0.776157	0.036945	−0.50997
*fhp*	PA14_29640	Nitric oxide dioxygenase	7,728	8,878	641,438	396,561.5	0.200139614	6.37507092	5.681305717
*chiC*	PA14_34870	Chitinase	138,470.5	132,918.5	3,525,171.5	3,615,357	−0.059036737	4.670042887	4.706487554
*hcnA*	PA14_36330	Hydrogen cyanide synthase	32,103.5	38,430	44,841	9,523	0.259502384	0.48208786	−1.753242554
*phzG2*	PA14_39880	Pyridoxamine 5'-phosphate oxidase	111,291.5	88,845	2,035,315	1,165,545	−0.324980918	4.192836779	3.388589391
*phzF2*	PA14_39890	Phenazine biosynthesis protein	230,024.5	139,410.5	1,982,482	1,048,278.5	−0.722448306	3.107448331	2.188162617
*phzE2*	PA14_39910	Phenazine biosynthesis protein	301,661	202,642.5	3,213,651.5	1,532,300.5	−0.57399141	3.41321339	2.344699156
*phzD2*	PA14_39925	Phenazine biosynthesis protein	188,527	137,242.5	2,063,245.5	977,075.5	−0.458043843	3.452072434	2.373698891
*phzC2*	PA14_39945	Phenazine biosynthesis protein	183,029	156,044	4,730,067	1,969,682.5	−0.230119369	4.69171646	3.427818936
*phzB2*	PA14_39960	Phenazine biosynthesis protein	211,229	178,704.5	4,517,504	1,991,168	−0.241231955	4.418646055	3.236735127
*phzA2*	PA14_39970	Phenazine biosynthesis protein	10,520.5	11,533	238,645	61,057.5	0.132564568	4.503590933	2.536965247
*mvfR*	PA14_51340	Transcriptional regulator	235,392	214,018	301,183	267,969	−0.13733315	0.355575051	0.187000822
*phnB*	PA14_51350	Anthranilate synthase component II	573,512.5	694,083	350,722	434,424	0.275283263	−0.709497	−0.400721123
*phnA*	PA14_51360	Anthranilate synthase component I	2,315,285.5	2,884,404	1,160,599.5	1,406,882.5	0.317083143	−0.996319892	−0.718688262
*pqsE*	PA14_51380	Quinolone signal response protein	2,517,240	3,188,208	1,210,331	1,483,216.5	0.340902981	−1.056441125	−0.763113575
*pqsD*	PA14_51390	3-Oxoacyl-ACP synthase	4,416,729.5	6,055,112.5	2,409,895	2,742,260	0.455175286	−0.874008189	−0.687613115
*pqsC*	PA14_51410	3-Oxoacyl-(acyl carrier protein) synthase	3,832,517.5	5,614,134	2,211,818.5	2,097,049	0.550771119	−0.793059375	−0.869931807
*pqsB*	PA14_51420	3-Oxoacyl-(acyl carrier protein) synthase	3,767,946.5	5,794,985	2,286,881.5	2,171,803.5	0.621026446	−0.72039687	−0.794884904
*pqsA*	PA14_51430	Coenzyme A ligase	2,644,556.5	3,743,059.5	1,089,687.5	870,485.5	0.501192183	−1.279111339	−1.603133627
*katB*	PA14_61040	Catalase	12,312.5	13,211.5	3,028,118.5	2,895,168	0.101670551	7.942154129	7.877379532

Many of the *phz* genes were exceptions to the above trend of WT or higher levels of gene expression in backgrounds that were hypersensitive to C_4_HSL. The enzymes responsible for the synthesis of pyocyanin are encoded by the *phzABCDEFG1/2* operons, *phzH*, *phzM*, and *phzS*. We previously mapped RhlI-dependent RhlR-binding sites upstream of the *phzA1*, *phzA2*, and *phzH* genes and within the *phzB1* and *phzB2* coding sequences (37). Thus, we hypothesized that these genes would be sensitive to the addition of C_4_HSL. Consistent with the above phenotypic analyses of pyocyanin production, *phz* gene expression was lower in strains of *P. aeruginosa* that expressed a hypersensitive RhlR variant. Notably, the expression of *phzA/B/C1* was especially sensitive, as gene expression levels were comparable to that of a Δ*rhlI* strain without exogenous C_4_HSL (Fig. 6B; Fig. S7E). A strain expressing T58L had lower *phz* gene expression across all of the *phz* genes, with the exception of *phzH*, which encodes the enzyme responsible for converting phenazine-1-carboxylic acid (PCA) to phenazine-1-carboxamide (PCN). All of the genes in the *phzABCDEFG1/2* operons were expressed at least 25% lower in a strain expressing the RhlR T58L variant compared to WT RhlR. To confirm our RNA-seq results of decreased phenazine gene expression, we performed chromosomal replacement of WT *rhlR* with our mutant *rhlR* alleles into *phz1*-mScarlet and *phz2*-mScarlet reporter *P. aeruginosa* strains (Fig. S7E). Interestingly, a strain expressing RhlR T58L had an insignificant decrease in *phz1*-mScarlet expression, while *phz2*-mScarlet expression decreased by 4.5-fold (Fig. S7E). Thus, the *phz2* promoter is likely more sensitive to increased C_4_HSL signaling. Conversely, both *phz* reporters displayed similar twofold decreases in a strain expressing RhlR A44M (Fig. S7E). We next measured *phz2*-mScarlet expression in the context of colony biofilms (Fig. S8A). These data were consistent with our observations of biofilm morphology and biofilm pyocyanin production (Fig. 5C and D); *phz2* reporter expression was maximal in the WT strain, significantly decreased in a strain expressing RhlR T58L, and moderately decreased in a strain expressing RhlR A44M (Fig. S8B). In total, these data show that RhlR sensitivity to C_4_HSL can be promoter specific and dependent on the environment. We note that *phzH*, *phzA1*, and *phzA2* have separate RhlR-binding sites in their respective promoter regions. Thus, RhlR sensitivity to C_4_HSL controls differential *phz* gene expression from the different promoters that contain RhlR-binding sites; hyperactivation of RhlR leads to lower levels of *phz* gene expression from the *phzA1* and *phzA2* promoters but not *phzH*.

To determine the relative levels of different phenazines produced as a result of the altered expression of the *phz* genes, we performed liquid chromatography-mass spectrometry to quantify pyocyanin, PCA, and PCN from planktonic cultures (Fig. 6C; Fig. S9). Consistent with our spectrophotometric data, pyocyanin levels were lower in a strain expressing RhlR T58L relative to WT RhlR. This decrease may be in part due to lower *phzABCDEFG1/2* and *phzS* in RhlR T58L relative to WT RhlR (Fig. 6B; Fig. S7E; Table 2). Given that *phzH* gene expression levels were higher in a strain expressing RhlR T58L compared to WT RhlR, we hypothesized that PCN, the product of PhzH activity, would be elevated in a strain expressing RhlR T58L. Indeed, PCN levels increased by 37% in a strain expressing RhlR T58L (Fig. 6C). We surmise that the strain expressing RhlR T58L maintains enough gene expression through the *phz1* and *phz2* operons that a sufficient amount of substrate is available for PhzH to synthesize PCN. Thus, we expected that the concentrations of PCA would remain relatively unchanged between WT and RhlR T58L strains and, indeed, WT and RhlR T58L produced nearly identical levels of PCA (Fig. 6C). Unlike the strain expressing RhlR T58L, the strain expressing RhlR A44M was deficient in the production of all phenazine metabolites (Fig. 6C), which is consistent with the phenotypic and transcriptional data that showed a complete loss of gene expression from all of the *phz* promoters (Fig. 5B and 6A). Thus, changes in gene expression can alter the flux of metabolites through the phenazine metabolic machinery, which alters the pool of secreted phenazines, resulting in lower levels of pyocyanin and higher levels of PCN.

### Decreased levels of PqsE lead to decreased promoter binding for RhlR at phenazine gene promoters

**Fig 7:**
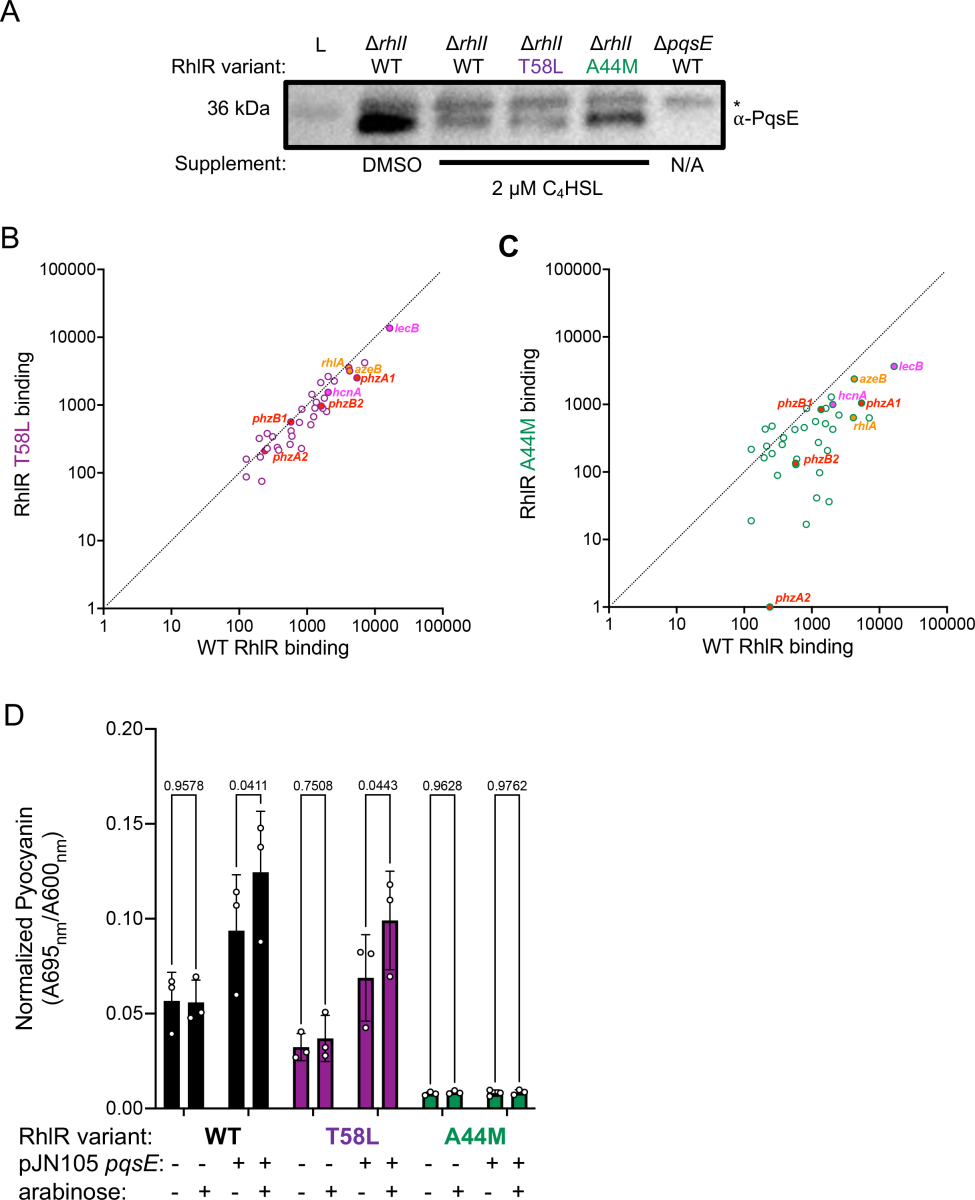
Repression of *pqsE* alters the ability of RhlR to bind to the *phz* promoter. (**A**) Western blot analysis depicting PqsE protein levels in the designated strains using a polyclonal antibody raised against purified PqsE. (**B**) Comparison of ChIP enrichment from the Δ*rhlI* and Δ*rhlI* RhlR T58L strains or (**C**) Δ*rhlI* RhlR A44M, all containing 2 µM of C_4_HSL. (**D**) Pyocyanin production was measured in WT *P. aeruginosa* strains expressing WT RhlR or the designated RhlR variants. Pyocyanin was quantified as pigment production (A695 _nm_) divided by cell density (A600 _nm_). Each strain contained a pJN105 empty vector (−) or pJN105 containing *pqsE* (+) under the control of the pBAD promoter. Arabinose was supplied at a final concentration of 0.1% (vol/vol) (+) or an equivalent volume of water (−) was supplied to all cultures. Statistical analyses were performed using an ordinary two-way analysis of variance with a Šídák’s multiple comparisons test. Comparisons between the uninduced (−) and the induced (+) are shown.

To determine the molecular basis for lower levels of *phz* gene expression in a background that expressed a hypersensitive variant of RhlR, we assessed expression of the *pqsABCDE* operon (51). PqsE is required for optimal expression of the *phz* genes and, thus, pyocyanin production. RhlR was previously characterized as a repressor of the *pqsABCDE* operon. We hypothesized that a C_4_HSL hypersensitive variant would repress *pqsABCDE* expression, which could lead to the observed decrease in *phz* gene expression. Indeed, *pqsA* RNA levels were 20% lower in a strain expressing RhlR T58L compared to WT RhlR (Fig. 6A; Table 2). To confirm that the decrease in gene expression led to a decrease in the available PqsE protein that was capable of interacting with RhlR to specifically drive it to the *phz* promoter, we performed western blot analyses to measure PqsE protein levels in strains expressing RhlR variants (Fig. 7A). PqsE levels were approximately twofold lower in a strain expressing the RhlR T58L variant compared to WT RhlR, indicating that, despite its hypersensitivity to C_4_HSL, RhlR requires a certain amount of PqsE to initiate transcription of the *phz* genes. We recently showed that PqsE dimerization is concentration dependent (46) and that it is required for complex formation (27). Thus, we hypothesized that when PqsE levels fall below a certain concentration, PqsE homodimerization cannot occur, thereby preventing PqsE-RhlR complex formation and reducing promoter occupancy. To determine if the lower levels of PqsE resulted in less enrichment of RhlR at RhlR-dependent promoters, specifically at the phenazine promoters, we performed ChIP-seq analyses to assess RhlR DNA-binding genome wide (Fig. 7B and C; Fig. S10A through C; Table 3). We highlight *phzA1* and *phzM*, which share a promoter (Fig. S10A), *rhlA* (Fig. S10B), and *lecB* (Fig. S10C) because of the role of the *phz* and *rhlA* promoters in regulating pyocyanin and rhamnolipid production (Fig. 5) and because *lecB* is a well-characterized PqsE-dependent promoter (37). As expected, RhlR T58L had lower promoter occupancy than WT RhlR at the *phzA1* promoter, likely due to the lower levels of PqsE present (Fig. 7B; Fig. S10A). Conversely, RhlR occupancy at the *rhlA* promoter was nearly unchanged from WT RhlR (Fig. 7B; Fig. S10B), reflecting the ability of RhlR to bind the promoter primarily through its dependency on C_4_HSL. Additionally, RhlR A44M occupancy was more significantly reduced at the *phzA1/phzM* and *rhlA* promoter, consistent with C_4_HSL binding being important for RhlR occupancy at these promoters (Fig. 7C; Fig. S10A and B).

**TABLE 3:** ChIP-seq analyses for all 40 previously annotated RhlR-binding sites^*a*^

Peak location	Synonym	Δ*rhlI* WT RhlR dimethyl sulfoxide	Δ*rhlI* RhlR A44M C_4_HSL	Δ*rhlI* WT RhlR C_4_HSL	Δ*rhlI* RhlR T58L C_4_HSL
64,211	*phzH*	1,326.683023	1,287.070428	1,937.052368	800.3602663
139,733	*PA14_01490* (*rahU*)	357.9828239	36.2904627	1,797.691814	1,262.330461
560,072	*PA14_06320*	795.6197881	207.8229081	1,705.160561	896.362682
754,615	*rpsL* operon	19.36026131	128.1334001	580.9222305	416.0978903
812,427	phzB1*, phzC1	1,630.745305	872.4500466	1,614.072175	968.0690828
813,529	*phzA1* operon, *phzM*	2,478.166203	1,047.872334	5,438.76762	2,515.768325
895,433	*PA14_10360*	677.6307237	557.9219438	1,134.931646	513.5021075
1,372,370	PA14_16100*, PA14_16110	175.1045666	41.26732953	1,180.851185	1,439.936588
1,387,361	*lasB*	80.33453449	155.0928863	597.9287268	347.7142159
1,620,628	*PA14_18800*	501.1176115	459.0169075	664.0795222	405.7600301
1,621,028	*PA14_18810* operon	197.2321883	216.7086261	127.1210219	87.20039346
1,648,391	*rhlA* operon	1,269.331414	635.9695744	4,107.630943	3,584.538326
1,651,804	*rhlI*	266.5774697	424.9503683	562.0829081	263.1137194
1,735,823	*PA14_20130, PA14_20140* (*fpr*)	99.86648863	187.1783447	258.1792167	380.5321279
1,774,336	*lecB*, *PA14_20620* operon	7,502.473941	3,659.463345	16,502.76243	13,604.76476
1,816,906	*PA14_21020* (*azeB*) operon, *PA14_21030*	2,255.468809	2,385.089908	4,251.598666	3,188.488032
1,921,598	*PA14_22090*	309.7522265	403.3132102	402.4723862	349.8223501
2,444,398	PA14_28250*, *PA14_28260*	5.80852955	18.90288673	126.8210075	159.5263335
2,568,048	*PA14_29620, fhp* operon	1,214.493077	698.1271296	2,516.668544	2,263.303886
2,647,472	*PA14_30570, PA14_30580* (*vqsR*)	332.4663954	454.8525009	770.6777749	554.7665595
2,677,542	PA14_30840*	2,532.338968	631.2317874	7,049.74026	4,222.321191
2,721,761	*pa1L*	397.191987	431.3729694	204.7856305	171.4111541
3,188,223	*tnpS, tnpT* operon	97.58852796	254.9923469	365.2390913	237.0982179
3,236,436	*hcnA* operon, *exoY*	1,975.895771	998.8226755	2,043.830658	1,543.720024
3,364,761	*PA14_37745* operon	33.84992184	0	408.8172656	433.4142868
3,561,157	phzB2*, phzC2	282.5987452	241.2144489	214.5461642	75.06140576
3,561,969	*phzA2* operon	266.3857949	134.5528845	575.2425938	562.6382415
3,600,666	*lasA*	49.28016756	0	239.3835631	211.7123794
3,831,541	*hsiA2* operon, *hcpD*	725.4822967	840.7603325	1,370.159237	1,100.666745
4,285,523	*PA14_48140* operon	250.461593	89.05541827	311.12285	340.9372559
4,313,855	*PA14_48530* operon	676.5816394	870.1911185	838.3261442	867.5673944
4,314,560	PA_48530*	304.6211444	476.7892079	258.8493931	229.0054027
4,382,169	*PA14_49310*	369.2669642	273.4166314	1,250.836498	673.3678123
4,425,570	*PA14_49740, PA14_49750* operon	392.3756839	518.7203442	1,588.805785	2,142.31475
5,159,548	PA14_57970*, *PA14_57980*	100.2024347	16.73202365	826.0484211	228.802281
5,268,881	PA14_59180*	103.8910684	320.1317375	381.3763735	216.4315472
5,414,219		52.20673959	86.35911849	519.0410351	697.0174452
6,082,366	*rmlB* operon	470.8855602	428.1094796	2,046.603614	2,633.971539
6,093,032	*arcD* operon	221.4399742	97.45924584	1,302.256041	900.8360611

^*a*^ "*" indicates internal binding site of that gene.

RhlR T58L did not have significantly altered occupancy at the *lecB* binding site compared to WT, which is counter to our expectations. We hypothesize that reduced PqsE levels would have an effect on RhlR binding to PqsE-dependent promoters (Fig. S10C). Furthermore, RhlR occupancy did not correlate with gene expression from these promoters, which we also observed when we previously performed ChIP-seq experiments in *rhlI* and *pqsE* deletion strains. For example, despite RhlR T58L and RhlR A44M occupying the *lecB* promoter at different levels, the expression profiles were nearly identical (Fig. S10C; Tables 2 and 3). While it was initially surprising to observe that RhlR T58L could occupy the *lecB* promoter because it is so strongly dependent on the levels of PqsE, we hypothesize that if some number of PqsE dimers exist in complex with RhlR, these complexes would be preferentially driven to the *lecB* promoter. The *lecB* promoter is one of the “preferred” sites for PqsE-RhlR binding in the genome, as determined by map reads to the *lecB* promoter from our previous ChIP-seq data (37). Additionally, we cannot rule out that the increased efficacy of C_4_HSL for RhlR T58L alters RhlR-binding site preferences. Future studies aimed at determining the hierarchy of RhlR promoter selection will be needed to confirm this assertion.

To test whether low levels of PqsE were directly leading to the dysfunction in the regulation of the phenazine genes in strains that were hyper-responsive to C_4_HSL, we overexpressed *pqsE* from the pBAD promoter on the pJN105 plasmid in WT, RhlR T58L, and RhlR A44M variant backgrounds of *P. aeruginosa* and assayed for pyocyanin production (Fig. 7D). All strains received the equivalent pJN105 empty vector control. As shown in Fig. 5, the strain expressing RhlR T58L that contained the empty plasmid produced less pyocyanin than the WT counterpart. We note that we observed an intermediate enhancement of pyocyanin production for the strains expressing WT RhlR and RhlR T58L containing pJN105-*pqsE*, likely reflecting the leaky expression of *pqsE* under the pBAD promoter (Fig. 7D). Nevertheless, induction of *pqsE* expression from the pBAD promoter with the addition of arabinose enhanced pyocyanin production in both the WT strain as well as the strain expressing RhlR T58L, as expected, indicating that levels of PqsE are important for driving and fine-tuning RhlR-dependent transcription (Fig. 7D). Last, the strain expressing RhlR A44M did not exhibit restored pyocyanin production under any of the conditions tested (Fig. 7D), consistent with the results from the *E. coli* reporter system (Fig. 3A and B) and the phenotypic assays performed in *P. aeruginosa* (Fig. 5). Thus, the RhlR A44M variant is unaffected by PqsE levels and represents a completely inactive form of RhlR. In total, RhlR promoter binding must be effectively coupled with RNA polymerase to drive gene expression, and this is likely mediated both by PqsE and allosteric effects induced by C_4_HSL occupancy in the RhlR LBP.

## DISCUSSION

**Fig 8:**
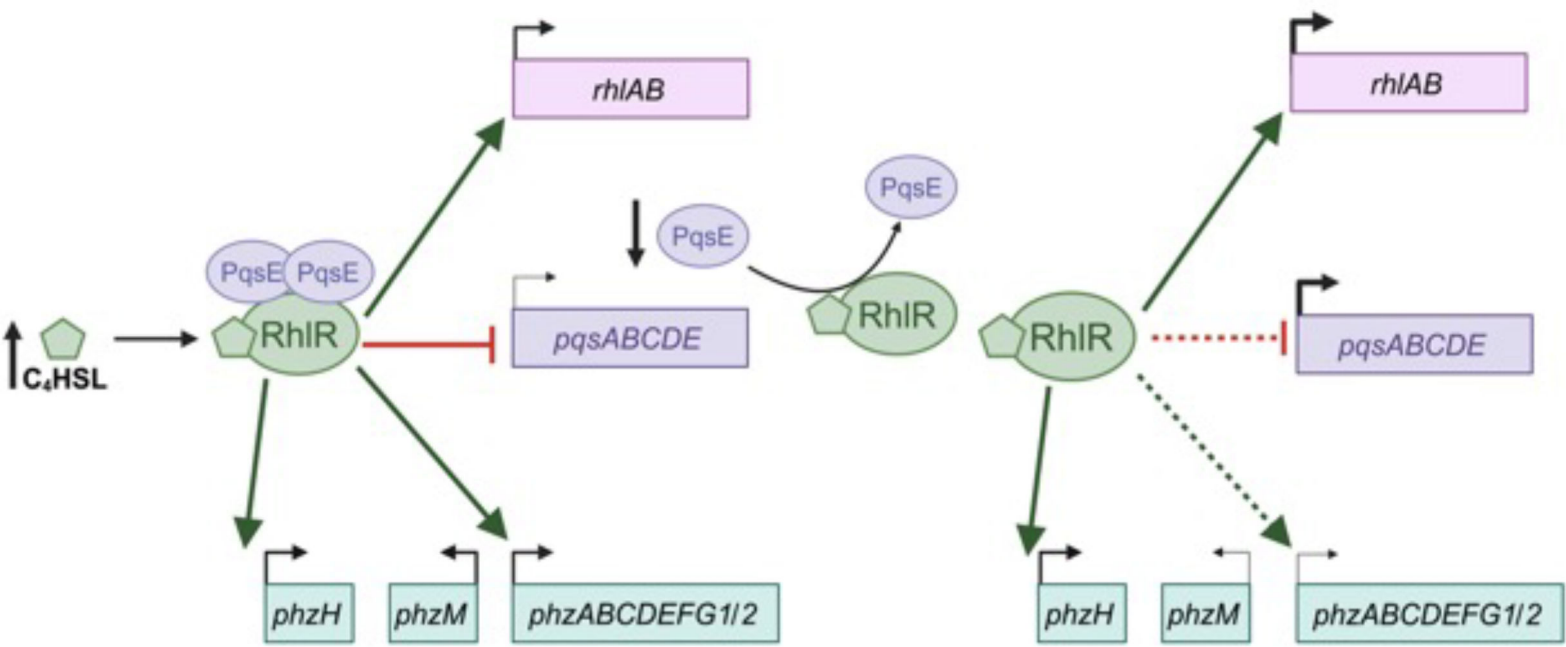
Model for C_4_HSL- and PqsE-dependent gene expression. The levels of C_4_HSL and PqsE tightly regulate the activation of RhlR by altering its binding to specific DNA sites.

Here, we showed that amino acid substitutions at T58 increase the sensitivity of RhlR to C_4_HSL, which can have significant disparate effects on downstream signaling. We hypothesize that the RhlR LBP reflects an evolutionarily optimized sensitivity to C_4_HSL. It is intriguing that RhlR has such a high EC_50_ value and requires >1 µM C_4_HSL for QS activation. This is in stark contrast to LasR, a second LuxR-type receptor also found in *P. aeruginosa*. This likely reflects the role of specificity and sensitivity of the two systems. LasR has a high sensitivity for 3OC_12_HSL, but it comes at the cost of decreased selectivity; LasR can be activated by 3OC_8_HSL, 3OC_10_HSL, and 3OC_14_HSL, albeit with reduced sensitivity relative to 3OC_12_HSL (29). We hypothesize that this is due to the placement of LasR in the QS hierarchy. LasR signaling is initiated prior to RhlR activation, and as an early sensor for the chemical signaling environment, it might be advantageous to have initial flexibility in AHL binding to robustly initiate QS to outcompete neighboring bacteria that produce non-cognate AHL. Conversely, RhlR sacrifices sensitivity for selectivity and appears to maintain this level of sensitivity under some evolutionary constraint, given that we show it could have evolved greater sensitivity. We hypothesize that this is due to the costs associated with producing phenazines (52). More specifically, the overproduction of pyocyanin can be toxic to *P. aeruginosa*. Thus, if RhlR were too sensitive to C_4_HSL and/or overly promiscuous, it would be difficult to fine-tune or maintain proper regulation of pyocyanin. Additionally, this fine-tuning is optimally achieved because RhlR has co-evolved with PqsE to control expression of its regulon. Thus, RhlR coincidence detection via C_4_HSL and PqsE underpins late QS behaviors in *P. aeruginosa* (Fig. 8).

We used RhlR hyperactive variants to mimic hyperactivation by C_4_HSL and to determine the crucial role of C_4_HSL sensing in QS progression. The phenazines are subject to complex regulation to allow for adaptation to different environmental conditions. Our data support the unique and dynamic role of RhlR-PqsE regulation; C_4_HSL levels influence both C_4_- and PqsE-dependent activation of different phenazine genes. Specifically, our data showed that an increase in *phzH* expression relative to *phzM* shuttles substrates to the production of PCN. PCN can be reduced and re-oxidized to function as an electron acceptor, thereby helping the cell maintain a balanced redox state (53, 54). This is especially important during anaerobic growth and biofilm growth; PCN enables the conversion of glucose and pyruvate to acetate to promote redox homeostasis and ATP synthesis, which is essential for cell survival during fermentative growth (52, 55). We hypothesize that C_4_HSL functions as a proxy for this “extreme” environment. During biofilm formation, we expect that C_4_HSL levels would be highest at the center of the biofilm, with the physical constraints of the biofilm dampening C_4_HSL diffusion into the greater extracellular milieu. In turn, this would signal to the cells to convert PCA to PCN rather than pyocyanin to ensure the survival of cells grown at the center of the biofilm. Meanwhile, cells at the periphery are actively growing, secreting, and sensing freely diffusible C_4_HSL, which subjects this population to the cycling coincidence detection observed in our RhlR-PqsE gene expression data and coincides with the robust production of pyocyanin instead of PCN. Thus, a single coincidence detection system can act as a switch to allow for the formation of different parts of a colony biofilm.

This mechanism, however, does not fully explain how *phz* reporter expression decreases at high levels of C_4_HSL in a heterologous *E. coli* expression system, as seen by others (45). When *pqsE* expression was driven by a non-native promoter and, thus, could not be repressed by the RhlR-C_4_HSL complex, *phzM* promoter-driven expression of light decreased. This is likely because high levels of both C_4_HSL and PqsE bound to RhlR have an inhibitory effect (35, 45). We hypothesize that there are two mechanisms by which this can occur. First, our previous work showed potential cryptic RhlR-binding sites internal to the *phzB1* and *phzB2* genes, suggesting that under high occupancy of the *phz* operons, RhlR could have a second, unfavorable-binding site that adds a second layer of regulation (37). Additionally, we suspect that at high levels of C_4_HSL, RhlR may oligomerize into higher-order structures that can no longer promote transcription. While not verified, this notion is supported by the broad peaks observed at many RhlR-binding sites in our ChIP-seq data, potentially reflective of multiple-binding sites. Indeed, RhlR has only 40 binding sites in the *P. aeruginosa* genome, with many of them existing within promoter regions, and if RhlR were stabilized, it could drive promoter-specific oligomerization that alters transcription. Since PqsE aids in the stability of RhlR, it is likely that PqsE bound to RhlR contributes to this effect. This sequestration of RhlR under extremely favorable conditions may function as an internal feedback loop to suppress unwanted pyocyanin production, which is toxic to *P. aeruginosa* at high concentrations. This notion agrees with data presented here showing that hyperactive RhlR variants, such as T58L, have a lower threshold for activating pyocyanin production.

Interestingly, hyperactivation of RhlR by C_4_HSL did not have a similar effect on genes involved in cellular detoxification, such as superoxide dismutase, heme oxygenase, nitric oxide reductase, and alkyl hydroperoxide reductase. These are enzymes that would be beneficial to collective cell survival alongside the differential production of phenazines. However, regulation of these genes appears to be indirect, yet through the response of RhlR to C_4_HSL, as the only difference between strains in our RNA-seq experiments was the expression of the hyperactive RhlR variant. Thus, it is intriguing to envision that C_4_HSL levels function as a proxy for the late stages of QS, with survival adaptation to environmental stresses and perturbations being paramount via changes in gene expression, especially in the context of biofilms.

Our chemical-genetic approach revealed important nuances about the RhlR LBP that will be informative for future medicinal chemistry approaches aimed at inhibiting the RhlR-C_4_HSL interaction. In particular, the RhlR A44M variant was insightful because it revealed a portion of the LBP that, when perturbed, could reduce or eliminate the activation of RhlR by C_4_HSL. Importantly, RhlR A44M was soluble in the presence of PqsE, which allowed us to recapitulate the RhlR-PqsE interaction *sans* activating ligand, which had previously been a state of RhlR that could not be purified. In a cellular context, RhlR-PqsE, while unliganded, was incapable of driving gene expression from any promoter. RhlR A44M could, however, still bind to promoters that were previously shown to be reliant on PqsE for binding. We hypothesize that the RhlR A44M variant induces an allosteric structural change that eliminates the ability of RhlR to engage with RNA polymerase. It was previously postulated that fully inhibiting RhlR function would require suppression of both C_4_HSL- and PqsE-dependent traits (1, 39). Here, we show that strains expressing RhlR A44M fail to activate C_4_HSL- and PqsE-dependent traits via a single change in the RhlR LBP. Thus, if the pocket surrounding A44 in the RhlR LBP can be targeted with a C_4_HSL-like mimic that occupies the area adjacent to A44 with a hydrophobic moiety, RhlR-dependent gene expression could be effectively abolished to levels comparable to that of a △*rhlR*, which would render *P. aeruginosa* non-pathogenic.

## MATERIALS AND METHODS

### Phylogenetic tree construction

Two separate lists of protein sequences were retrieved from the National Center for Biotechnology Information: one for LuxR receptors and another for their partner LuxI synthases. Each list was aligned using MUSCLE v3.8.1551 and trimmed with trimAl v1.4.1 using default parameters. Maximum-likelihood trees were generated from alignments of 22 sequences for each group, with bootstrap support values calculated with 1,000 replicates using IQ-TREE v1.6.12. Model selection was performed using an automatic substitution model based on the Bayesian Information Criteria score, selecting the LG + G4 model for the LuxR tree and the LG + I + G4 model for the LuxI tree. The trees were visualized and annotated with Interactive Tree of Life v6.9.1.

### Plasmid and strain construction

Plasmids and strains were constructed using standard molecular cloning techniques. Specifically, *rhlR* in the pBAD-A plasmid was mutagenized using overlap extension PCR with site-directed mutagenesis primers designed using the Agilent QuikChange Primer Design Tool. Sequence-confirmed pBAD-A-*rhlR* mutants were transformed into an *E. coli* strain already containing pCS26-p*rhlA-luxCDABE* and pACYC-*pqsE* or the pACYC empty vector control. We also note that the RhlR and PqsE co-expression construct (pETDuet-1) was cloned to not include RhlR with an N- or C-terminal tag; PqsE was tagged at the N-terminus as it was previously shown to be functional *in vivo*, indicating that it has no effect on the ability of PqsE to bind to RhlR. Table S2 lists the plasmids and strains used in the study. Table S3 lists the primers used in the study.

### *P. aeruginosa* strain construction

Standard cloning and molecular biology techniques were used to generate *P. aeruginosa rhlR* mutations. Introduction of genes encoding RhlR variants onto the *P. aeruginosa* chromosome was achieved using previously published protocols (47). In brief, the entire *rhlR* mutant coding sequence was amplified, digested, and ligated into the pEXG2 vector. The pEXG2 vector containing *rhlR* mutants was then transformed into *E. coli* SM10 λ*pir*, followed by conjugation into the appropriate UCBPP-PA14 *P. aeruginosa* strain background. All strains were confirmed by amplifying the target locus followed by Sanger sequencing. Transcriptional reporters for the *phz1* and *phz2* operons were generated as previously described, with plasmids generously gifted by Dr. Lars Dietrich (56).

### *E. coli* light assay

All *E. coli* strains in this study were grown overnight with shaking in 5 mL Luria-Bertani (LB) media supplemented with 100 µg/mL ampicillin, 50 µg/mL kanamycin, and 15 µg/mL tetracycline at 37°C overnight. Overnight cultures were diluted 1:100 into fresh media, grown at 37°C with shaking for 2 h until reaching OD_600_ = 0.4. Cultures were induced with a final concentration of 0.1% arabinose, and 198 μL of the induced culture was mixed with 2 μL of C_4_HSL dilutions in a clear-bottom, black-opaque 96-well plate (Corning) and grown at 37°C with shaking for 4 h. The C_4_HSL dilution series was performed 1:1 in dimethyl sulfoxide (DMSO) with a top final concentration of 10 μM. Luminescence and optical density were recorded using a Glomax (Promega) plate reader.

### WT RhlR, RhlR variant, and PqsE protein expression, affinity purification pulldown, and large-scale purification

To determine protein solubility ± AHL or PqsE, WT RhlR and other RhlR variant genes were cloned into the multiple cloning site 2 of the pETDuet-1 vector using NEBuilder HiFi Assembly cloning kit. To perform the affinity purification pulldown, the 6x-His-*pqsE* gene was cloned using the same cloning method into the multiple cloning site 1 of pETDuet-1, already having either WT *rhlR* or *rhlR* mutant genes in the multiple cloning site 2. Sequence-confirmed plasmids were transformed into *E. coli* BL21 (DE3) cells. For both solubility and pulldown assays, overnight cultures were back diluted 1:100 in 25 mL LB supplemented with 100 µg/mL ampicillin and grown at 37°C to an OD_600_ = 0.6. The cultures were induced using 0.3 mM IPTG and 200 µM C_6_HSL and incubated at 18°C with shaking at 130 rpm for 8 h. The cells were harvested at 4,500 rpm for 10 min at 4°C and re-suspended in the ice-cold lysis buffer containing 50 mM Tris-HCl pH 8.0, 150 mM NaCl, 0.1% Triton X-100, and 7 mM imidazole. Cells were lysed using probe sonication (20% power, 15 sec on/off pulses for 3 min), and the cell lysates were spun at 14,000 rpm for 30 min at 4°C to collect the soluble fraction. To check the solubility of WT or RhlR variants, whole cell lysates and soluble fractions were analyzed using SDS-PAGE. For affinity purification pulldowns, Ni-NTA magnetic beads (New England Biolabs) were incubated with soluble protein fractions. A total of 30 µL pre-equilibrated magnetic beads with lysis buffer were added to 1 mL of each soluble fraction (input) and incubated at 4°C for 45 min with mixing. The protein-bound beads were washed with 20-column volume of wash buffer (50 mM Tris-HCl pH 8.0, 150 mM NaCl, 0.1% Triton X-100, and 30 mM imidazole). The bound protein complexes were collected using elution buffer (50 mM Tris-HCl pH 8.0, 150 mM NaCl, 0.1% Triton X-100, and 300 mM imidazole; output). To evaluate the interaction between PqsE and WT RhlR or RhlR variants, both input and output fractions were analyzed using SDS-PAGE. Gels were stained using Coomassie brilliant blue and imaged on a BioRad EZ-Doc gel imager.

For large-scale purifications, 1 L of culture was used. RhlR and RhlR A44M were co-purified with 6x-His-PqsE using the same protocol described for the affinity purification pulldown method. The cells were harvested at 8,000 rpm at 4°C, resuspended in 40 mL of lysis buffer, and then lysed using probe sonication at 20% amplitude with a 15 s on/off cycle for 15 min on ice. The cell lysate was spun at 14,000 rpm at 4°C for 45 min to remove the cell debris. The supernatant was collected and then mixed with 1 mL of Ni-NTA beads (Qiagen) that were pre-equilibrated with lysis buffer. The slurry was incubated at 4°C for 45 min. The protein-bound beads were washed with 15 mL of wash buffer, and the protein was eluted using the elution buffer. Ni-NTA fractions containing PqsE and RhlR were concentrated and further purified using a Superose-6 column (Cytiva) pre-equilibrated with 50 mM Tris HCl pH 8.0, 150 mM NaCl, and 200 µM C_6_HSL. Protein purity was checked via SDS-PAGE. The gel was stained with Coomassie brilliant-blue and imaged on a Bio-Rad EZ-Doc gel imager.

### Pyocyanin production assay

For Fig. 5A, overnight *P. aeruginosa* cultures were grown in 3 mL LB at 37°C. Pyocyanin was measured as previously described (34). For Fig. 5B, overnight *P. aeruginosa* cultures were grown and back diluted 1:100 in 20 mL LB containing 2 μM C4HSL or a DMSO carrier control in strains lacking *rhlI*. Back dilutions were grown for 5 h at 37°C, and pyocyanin was measured as previously described. For Fig. 5C, 3 µL of overnight *P. aeruginosa* cultures were spotted on Congo red plates and grown for 5 days in the dark at room temperature. Pyocyanin was extracted from the entire plate using chloroform, then extracted into 0.1 M HCl for quantification, and measured at 520_nm_ (57). For Fig. 6D, pyocyanin production assays were performed by measuring pyocyanin from planktonic culture supernatants. The PA14 WT and strains containing RhlR variants T58L and A44M were transformed with the pJN105 vector with or without the *pqsE* gene controlled by the pBAD promoter. The overnight cultures were washed and resuspended in LB media containing gentamycin (30 µg/mL) for plasmid selection, and the secondary cultures were started at an A600_nm_ = 0.1. The cultures were incubated at 37°C with shaking at 200 rpm. At A600_nm_ = 0.6, the cultures were induced with a final concentration of 0.1% (vol/vol) arabinose. Cultures were incubated for 10 h, and the culture supernatants were harvested by centrifugation. The cell-free supernatant was measured at A695_nm_.

### Swarming assay in *P. aeruginosa*

Swarming plates were made with a 5× M9 salt stock solution consisting of 2.7 g NH_4_Cl, 4.25 g Na_2_PO_4_, 7.5 g KH_2_PO_4_, and 1.25 g NaCl in 500 mL deionized water (58). A total of 40 mL 5× M9 stock was added to 150 mL deionized water with 0.8 g agar for a final agar concentration of 0.4%. A total of 200 µL of filter-sterilized 1 M MgSO_4_, 200 µL filter-sterilized 1 M CaCl_2_, and 2.7 g dextrose in 10 mL 0.5% casamino acids were used to obtain a total concentration of 10%. A total of 5 mL of overnight cultures was grown and normalized to an OD_600_ = 1. A total of 5 µL of normalized cell cultures was spotted on the center of the plate and left to dry. Plates are incubated face down at 37°C for 24 h and for another 12 h at room temperature. Images were taken using an Epson Perfection V850 Pro scanner at a resolution of 300 dpi and a display gamma of 2.2.

### Rhamnolipid production assay in *P. aeruginosa*

Clear 96-well plates (CellTreat) were dyed using 50 µL of 0.1 mg/mL of Victoria Pure Blue BO in isopropanol (59). The isopropanol was evaporated under a vacuum without spinning for 1 h at 45°C. A total of 300 µL of 0.5 M NaOH was added to the wells to fix the dye and incubated at room temperature for 10 min. The NaOH was aspirated, and the plates were dried under a vacuum without spinning for 1 h at 45°C. A total of 5 mL of overnight cultures was grown and back diluted into 1 mL cultures grown in 24-well plates (Greiner Bio-One) for 24 h. One milliliter of supernatant was collected after spinning the plate for 2 min at 15,060 rpm. In triplicate, 250 µL supernatant and a rhamnolipid control were added to dyed wells and incubated with shaking at 750 rpm for 1 h at room temperature. A total of 200 µL was transferred to a clean 96-well plate (CellTreat), and absorbance was read at 625_nm_ with a 5 s shake before reading (Molecular Devices SpectraMax M5 SoftMax Pro5).

### Congo red biofilm assay and *phz2* reporter expression in biofilms

Biofilm plates were made with 1% tryptone including 15.2 µM NH_4_SO_4_, 0.36 µM FeSO_4_, 1 mM KH_2_PO_4_ monobasic, 49 µM Congo red dye ([wt/vol]; Sigma Aldrich), and 12.5 µM Coomassie blue dye ([wt/vol]; Sigma Aldrich). Agar was added to a final concentration of 1%. One millimolar filter-sterilized MgSO_4_, 0.2% (wt/vol) Casamino acids, and 0.5% (wt/vol) glycerol were added to the cooled agar mixture after autoclaving prior to pouring. A total of 5 mL of overnight cultures expressing the *phz1/2* mScarlet transcriptional reporters were grown, and 3 µL were spotted at the center of the plate and left to dry. Plates were incubated face down in an air-tight box to control for humidity at room temperature in the dark for 5 days. Brightfield and red fluorescent images were taken after 5 days.

### Image analysis

#### Swarming quantification

Traces on swarming plate images were segmented through manual thresholding (0–255), based on pixel value intensities of each image. For swarming distance analysis, from the user-defined center point (*x*_0_, *y*_0_), the farthest distance traveled was calculated as the maximum value of distances of all boundary points to the center point (*r* = $\sqrt[{2}]{{\left(x-x_{0}\right)}^{2}+{\left(y-y_{0}\right)}^{2}}$).

#### Biofilm fluorescence signal analysis

Intensities of colony biofilms expressing the *phz1/2* mScarlet transcriptional reporters grown on Congo Red plates were corrected to WT UCBPP-PA14 non-fluorescence controls at each time point. For each corrected image, the sum of fluorescence intensity is calculated as $\sum_{}^{}Pixel\;Value$. Preprocessing and calculation were carried out with functions in the scipy and cv2 packages, see https://github.com/biqingliang/Structural-Basis-for-C4-selection-by-PA14-RhlR for deposited scripts.

### Western blot analysis

#### E. coli

Strains were grown and induced as described previously (see *E. coli* Light Assay), with a culture size of 15 mL. Bacterial pellets were collected by centrifugation at 10,000 rpm for 10 min at 4°C and stored at −20°C until processed. Cell pellets were lysed in 1% SDS, 150 mM NaCl, and 50 mM Tris pH 8.0 for 5 min and subsequently sonicated for 15 s at 10% amplitude three times. Whole cell lysate was then subjected to Pierce BCA Protein Assay Kit (ThermoFisher) to determine protein concentration. Loading dye (250 mM Tris pH 6.8, 8% SDS, 40% glycerol, and 8% 2-mercaptoethanol) was added to the lysates and boiled at 100°C for 5 min. Protein was loaded at a concentration of 20 µg/well into 4%–20% Mini-PROTEAN TGX Precast Protein Gels (Bio-Rad) and run in a Mini-PROTEAN Tetra Vertical Electrophoresis Cell (Bio-Rad) at 200V for 45 min. Gels were transferred to activated (100% methanol, 1 min) polyvinylidene fluoride (PVDF; Millipore Sigma) using a Trans-Blot SD Semi-Dry Transfer Cell (Bio-Rad). Total protein images were acquired using a ChemiDoc MP (Bio-Rad). Membranes were blocked in 5% dry milk powder in TBST for 1 h. Membranes were then incubated overnight in 5% milk in TBST with 1:1,000 α-RhlR (10) and washed three times for 5 min in TBST prior to the addition of goat α-rabbit-HRP secondary (1:10,000, Sigma). A chemiluminescent signal was detected using Pierce ECL Western Blotting Substrate (ThermoFisher) on a ChemiDoc MP. Band densitometry analysis was done in ImageJ (version 1.54m) (60) using the total protein image for normalization.

#### P. aeruginosa

Strains were grown with or without AHL in a culture size of 20 mL until reaching an OD_600_ = 2.0. For the PqsE western blots, bacterial pellets were collected by centrifugation at 10,000 rpm for 10 min at 4°C. Cell pellets were lysed in 150 mM NaCl, 50 mM Tris pH 8.0, and 20 mM imidazole and sonicated for 15 s at 25% amplitude three times. Whole cell lysate was then subjected to Pierce BCA Protein Assay Kit (ThermoFisher) to determine protein concentration. For the RhlR Westerns, loading dye (250 mM Tris pH 6.8, 8% SDS, 40% glycerol, and 8% 2-mercaptoethanol) was added to the lysates and boiled at 100°C for 10 min. Protein was loaded at a concentration of 20 µg/well into 4%–20% Mini-PROTEAN TGX Precast Protein Gels (Bio-Rad) and run in a Mini-PROTEAN Tetra Vertical Electrophoresis Cell (Bio-Rad) at 35 mA for 35 min. Gels were transferred to activated (100% methanol, 1 min) PVDF (Millipore Sigma) using a Trans-Blot SD Semi-Dry Transfer Cell (Bio-Rad). Membranes were blocked in 5% dry milk powder in TBST for 1 h. Membranes were then incubated overnight in 5% dry milk powder in TBST with 1:1,000 α-PqsE. Membranes were washed three times for 15 min in TBST prior to the addition of goat α-rabbit-HRP secondary (1:10,000, Sigma) in 5% dry milk powder in TBST. A chemiluminescent signal was detected using Pierce ECL Western Blotting Substrate (ThermoFisher) on a ChemiDoc MP.

### *rhlA* promoter-fusion activity assay in *P. aeruginosa*

The *rhlA* promoter mScarlet fusion construct was cloned in pUCP18 using restriction enzyme cloning (see Table S2). The construct was electroporated in PA14 WT, Δ*rhlI*, and Δ*rhlR* strains. Primary overnight cultures were grown at 37°C, and secondary cultures were back diluted 1:100 in 5 mL LB media supplemented with 400 µg/mL carbenicillin. The cultures were incubated for 24 h at 37 °C with shaking at 200 rpm. One milliliter of culture was pelleted at 3,000 rpm for 5 min. The supernatant was discarded, and the pellet was resuspended in 1 mL of 1× PBS. Fluorescence was recorded using a Glomax (Promega) plate reader with excitation at 520 nm and emission at 540–580 nm in a clear-bottom, black-opaque 96-well plate (Corning).

### RNA extraction and RNA-seq data analysis

#### RNA extraction

A total of 5 mL of overnight cultures was back diluted 1:100 in 20 mL of fresh LB media and incubated for 5 h at 37°C with shaking. Cells were harvested by centrifugation, and pellets were frozen until RNA extraction. RNA was extracted using the Invitrogen PureLink RNA Mini Kit. Pellets were resuspended in 100 µL lysozyme solution containing 10 mM Tris-HCl pH 8.0, 0.1 mM EDTA, and 1 mg/mL lysozyme. Five microliters of 1% SDS was added, and the solution was mixed with a vortex and incubated at room temperature for 5 min. A total of 350 µL lysis buffer (Invitrogen) was added with 10 µL 2-mercaptoethanol for each 1 mL of lysis buffer and vortexed. Cells were lysed by bead beating (BioSpec 0.1 MM Zirconia/Silica Beads) for two rounds of 50 s. Lysed cells were transferred to fresh microcentrifuge tubes with 250 µL of 100% ethanol and mixed with a vortex. Samples were spun in spin cartridges (Invitrogen) at 12,000 × *g* for 15 s at room temperature, and the flow-through was discarded. A total of 700 µL Wash Buffer I (Invitrogen) was added and spun at 12,000 × *g* for 15 s at room temperature, and the flow-through was discarded. A total of 500 µL Wash Buffer II (Invitrogen) with ethanol was added and centrifuged for 12,000 × *g* for 15 s at room temperature; the flow-through was discarded, and RNA samples were washed again. Cells were spun at 12,000 × *g* for 1 min at room temperature to dry the membrane with the bound RNA. A total of 50 µL RNAse-free water was added to the center of the spin cartridge, incubated at room temperature for 1 min, and centrifuged for 2 min at ≥12,000 × *g* at room temperature. Extracted RNA was then frozen at −80°C until further use.

#### Data analysis

Bulk RNA was sent to SeqCenter (Pittsburgh, PA) where it was processed for paired-end Illumina sequencing via rRNA depletion using Ribo-Zero Plus (Illumina). Fastq files generated by SeqCenter were processed using Rockhopper with default settings (61–63). Briefly, fastq files were aligned to the genome of *Pseudomonas aeruginosa* UCBPP-PA14. The reads were subsequently normalized using upper-quartile normalization, and transcript abundance was determined by reads per kilobase per million mapped reads. Differential gene expression analysis was conducted using a negative binomial distribution model, outputting a *P*-value for each transcript. The *P*-values were then corrected for false discovery using the Benjamini-Hochberg procedure, resulting in the *q*-values reported here (Table S2). SignalMap v2.0 (NimbleGen Systems) was used to visualize read counts for *phzABCDEFG1/2*, *phzH*, *phzM*, and *phzS* (Fig. 5). Aligned sequences generated in Rockhopper were converted to GFF files, and genes of interest were isolated into unique GFF files (37).

### Liquid chromatography mass spectrometry of QS metabolites

#### Molecule extraction

A total of 5 mL overnight cultures were back diluted 1:100 in 20 mL fresh LB media containing 2 μM C_4_HSL or a DMSO carrier control in strains lacking *rhlI*. Back dilutions were grown for 5 h at 37°C, and supernatants were harvested by centrifugation and frozen until use. Sample supernatants were thawed, and 200 μL were added to a new 1.5 mL centrifuge tubes. Samples were extracted with 800 μL methanol, vortexed for 15 s, and centrifuged for 2 min at 10,000 × *g*. Samples were mixed 1:1 with 0.1% formic acid in a high-performance liquid chromatography vial and vortexed again.

#### Analytical measurements and analyses

An ultra-high-performance liquid chromatography (UHPLC) system coupled with a high-resolution mass spectrometer was employed for analyte quantification. The analytical platform consisted of a Vanquish UHPLC system interfaced with a Thermo LTQ Orbitrap mass spectrometer (Thermo Fisher Scientific), operating in positive electrospray ionization mode. Chromatographic separation was performed on a Hypersil GOLD C18 column (50 × 2.1 mm, 1.9 μm particle size; Thermo Fisher Scientific) using mobile phase A (0.1% [vol/vol] formic acid with 5 mM ammonium formate in water) and mobile phase B (0.1% [vol/vol] formic acid with 5 mM ammonium formate in methanol). The flow rate was set at 0.45 mL/min, and the column temperature was maintained at 45°C. The gradient elution program started at 5.0% B (held for 1 min), increased linearly to 100% B over 3.5 min, held at 100% B for 1 min, then returned to 5.0% B over 0.1 min, and held for an additional 1.4 min to re-equilibrate the column. The injection volume was 10 μL. To enable accurate quantification, a series of external controls at varying concentrations were run in the same batch with the unknown samples to generate a calibration curve. Unknown sample signals were normalized to this calibration curve to correct for batch-specific variability and instrument response drift. Quantification was performed using a 0.01 Da accurate mass window for peak integration. All data were acquired and processed using Xcalibur software (version 2.1, Thermo Fisher Scientific).

### ChIP sequencing

*P. aeruginosa* strains were grown overnight, back diluted 1:100 in fresh LB media, and grown to an OD_600_ = 2.0. Crosslinking, lysis, and immunoprecipitation were performed as previously described (37).

### Chromatin immunoprecipitation-qPCR

A total of 2 mL overnight cultures were back diluted 1:100 in 20 mL fresh LB media and incubated at 37°C with shaking until they reached an OD_600_ = 2. Cells were crosslinked by adding 500 µL formaldehyde for a final concentration of 1%, were swirled to mix, and left for 20 min. Crosslinked cultures were transferred to 50 mL conical tubes and quenched with 10 mL 2.5 M glycine for a final concentration of 0.5 M. Cells were pelleted at 4,000 rpm for 5 min, and the supernatant was discarded. Pellets were resuspended in 20 mL 1× TBS and spun at 4,000 rpm for 5 min. The supernatant was discarded, the pellet resuspended in 1 mL 1× TBS, and the cells were transferred to a microcentrifuge tube. Cells were spun at 14,000 rpm for 5 min, the supernatant was discarded, and the cell pellets were frozen until further use. Thawed cells were resuspended in 1 mL 150 mM FA Lysis Buffer (1 M HEPES, pH 7.5, 2 M sodium chloride, 0.5 M EDTA, 100% Triton-X 100, 5% sodium deoxycholate, 10% SDS, and water) containing 4 mg/mL lysozyme (Sigma) and incubated at 37°C for 30 min to lyse cells. Lysed cells were then sonicated at 0°C–10°C for 30 min with 30 s on/off pulses at 75% output with a BioRuptor Sonicator (Diagenode). Cell lysates were centrifuged twice for 10 min at 14,000 rpm. Between each centrifugation, the supernatant was transferred to new microcentrifuge tubes. One milliliter of cell lysate supernatants was diluted in 1 mL of 150 mM FA Lysis Buffer for a final volume of 2 mL. A total of 100 µL cross-linked, sonicated lysates were diluted in 800 µL 150 mM FA Lysis Buffer, and 20 µL were saved as input samples. A total of 30 µL of a 50% slurry of protein A beads in 1× TBS (Sigma) and 10 µL RhlR antibody were added to lysates and incubated at room temperature for 90 min on a rotisserie rotator. The immunoprecipitation mix was centrifuged for 1 min at 4,000 rpm, the supernatant was removed by aspiration, and the pellet was resuspended in 750 µL 150 mM FA Lysis Buffer. This mix was transferred to a Spin-X column (Corning), incubated for 3 min at room temperature on a rotisserie rotator, spun again for 1 min at 4,000 rpm, and the supernatant was removed by aspiration. This was repeated with 700 µL 150 mM FA Lysis Buffer, 500 mM FA Lysis Buffer (1 M HEPES, pH 7.5, 2 M sodium chloride, 0.5 M EDTA, 100% Triton-X 100, 5% sodium deoxycholate, 10% SDS, and water), ChIP Wash Buffer (1 M Tris-HCl, pH 8.0, 1 M lithium chloride, 0.5 M EDTA, NP-40, and 5% sodium deoxycholate), and TE (10 mM Tris-HCl pH 8.0 and 1 mM EDTA). After the last wash, protein A beads were resuspended in 100 µL ChIP Elution Buffer (1 M Tris-HCl pH 7.5, 0.5 M EDTA, and 10% SDS). The Spin-X column was placed in a fresh dolphin-nosed tube (Sorenson) and incubated for 10 min at 65°C with occasional gentle mixing. Immunoprecipitation mixes were centrifuged for 1 min at 4,000 rpm, and the Spin-X filters were discarded. Cells and input samples were de-crosslinked by incubating for 10 min at 100°C in microcentrifuge tubes. DNA was cleaned up with a PCR purification kit (Qiagen). Input samples were diluted 1:100 for 10 µL qPCR reactions. qPCR reactions were set up with 5 µL SYBR Select Master Mix (ThermoFisher), 2 µL cleaned DNA, and 3 µL combined forward and reverse primers. qPCR reactions were run with a 50°C holding stage, a 95°C holding stage, 40 cycles of 95°C–60°C, and a continuous melt curve stage from 95°C to 60°C (Applied Biosystems 7500 Fast Real-Time PCR System).

### Electrophoretic mobility shift assays

EMSAs were performed with promoter regions of *rhlA* and *phzA1* genes as previously described with minor modifications. To obtain the respective DNA fragment, 320 bp upstream of the *rhlA* gene and 723 bp upstream of the *phzA1* gene were PCR amplified from the genomic DNA of *P. aeruginosa* PA14 strain. Concentrations of PqsE, RhlR:C_6_HSL bound to PqsE, and RhlR A44M:C_6_HSL bound to PqsE were normalized per reaction. The EMSA reaction contained 1 µL of 10 ng/µL of the promoter DNA fragment, 4 µL of protein dilution (0.9375 µM, 1.875 µM, 3.75 µM, 7.5 µM, and 15 µM), and 15 µL of EMSA buffer (20 mM Tris-HCl pH 7.5, 30 mM NaCl, 0.5 mM EDTA, 0.2 mg/mL BSA, 10% glycerol, 1 mM DTT, and 10 mM MgCl_2_). Reactions were mixed by vortex and incubated at 30°C for 15 min. A total of 8 µL of the completed reaction was mixed with Novex Hi-Density TBE 5X Sample Buffer (Invitrogen) and loaded on an 8% polyacrylamide gel. Electrophoresis was conducted in a 1× TBE buffer at 100 V for 60 min at 4°C. The gels were washed using a 0.5× TB buffer. The gels were stained using 1× gel red (GoldBio) in 50 mL of 0.5× TB buffer with shaking, followed by three washes with 50 mL 0.5× TB buffer. Imaging was performed on Bio-Rad EZ-Doc gel imager using a UV sample tray capable of capturing emission spectrum of 550–700 nm (Bio-Rad) compatible with GelRed DNA staining dye.

## Data Availability

Sequencing data have been deposited at the NCBI Sequence Read Archive under the submission number PRJNA1338225. Code related to the imaging analyses performed in the manuscript can be found at https://github.com/biqingliang/Structural-Basis-for-C4-selection-by-PA14-RhlR.

## SUPPLEMENTAL MATERIAL

Supplemental figures jb.00013-26-s0001.pdf Figures S1 to S10.

Supplemental legends jb.00013-26-s0002.docx Legends for Figures S1 to S10.

Table S1 jb.00013-26-s0003.xlsx RNA-seq reads for all genes.

Table S2 jb.00013-26-s0004.docx Strains and plasmids used in this study.

Table S3 jb.00013-26-s0005.docx Primers used in this study.

ASM does not own the copyrights to Supplemental Material that may be linked to, or accessed through, an article. The authors have granted ASM a non-exclusive, world-wide license to publish the Supplemental Material files. Please contact the corresponding author directly for reuse.
