# Supplementary material for: RhlR quorum-sensing receptor ligand sensitivity regulates the differential expression of phenazine genes in Pseudomonas aeruginosa
Source: J Bacteriol. 2026 Apr 8;208(5):e00013-26. doi: 10.1128/jb.00013-26 (PMC13192270; doi:10.1128/jb.00013-26)
Supplement: Supplemental figures — Figures S1 to S10. [file jb.00013-26-s0001.pdf]

Figure S1

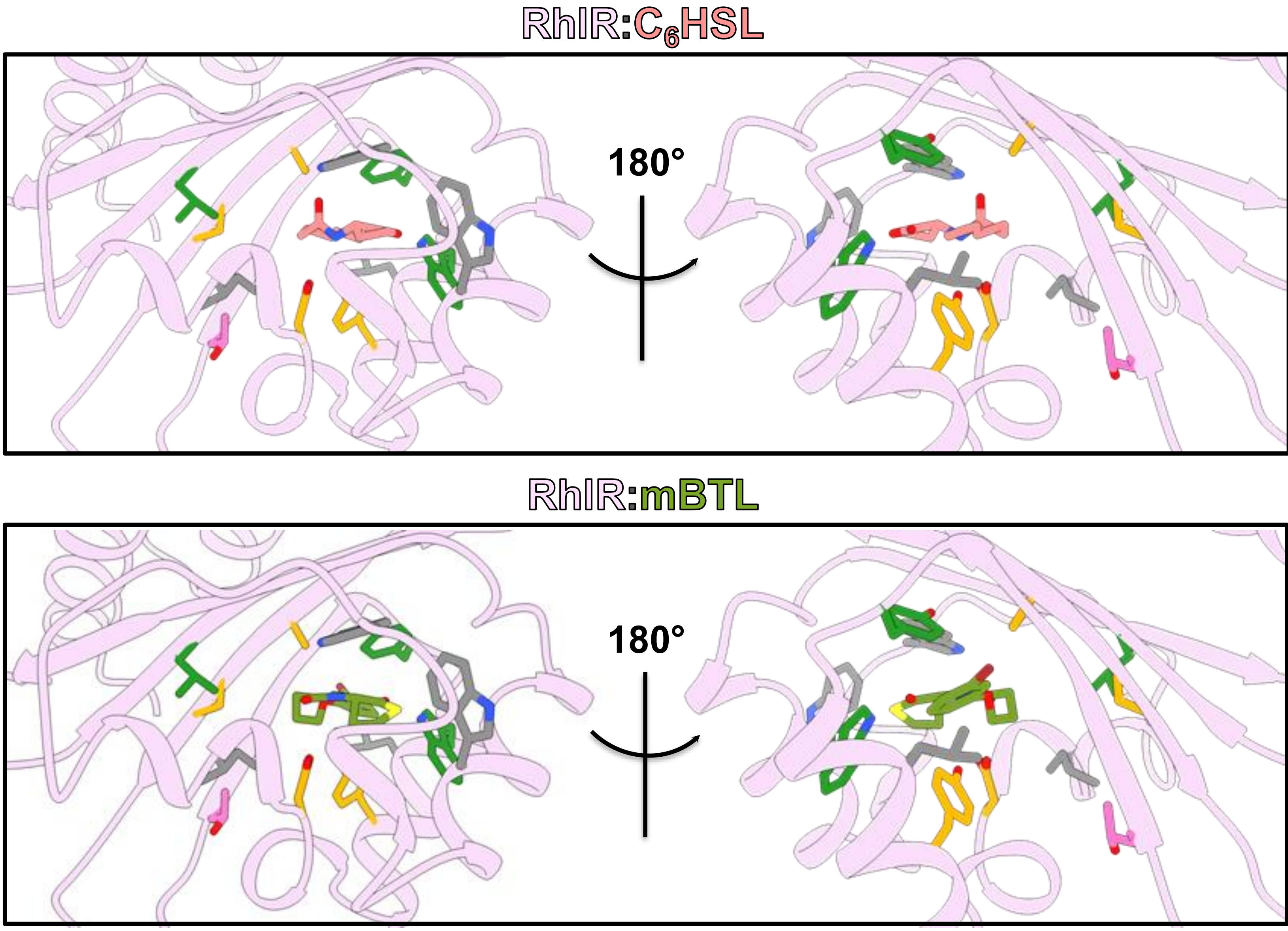

Figure S2

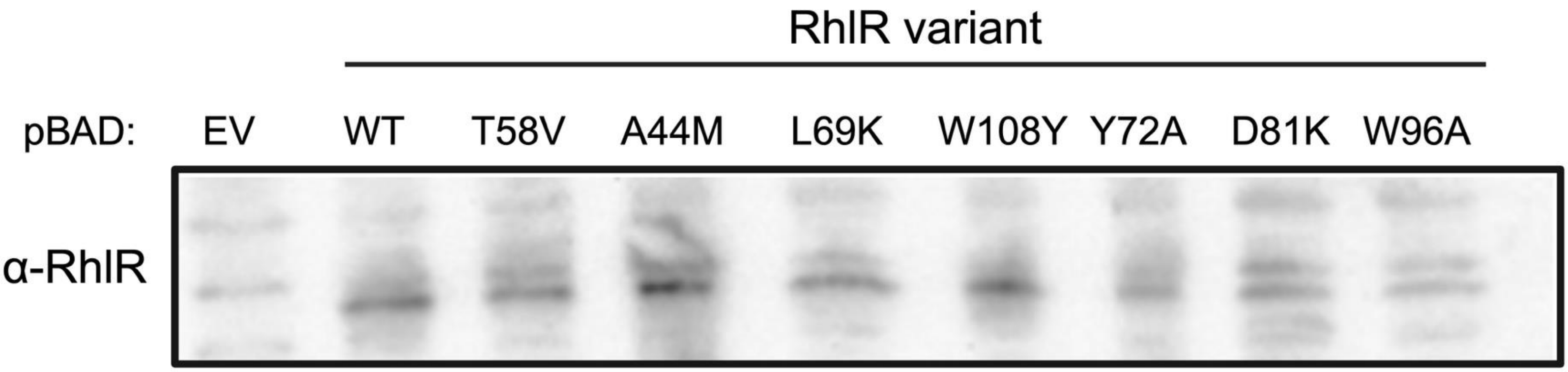
RhlR variant
pBAD:
EV
WT
T58V
A44M
L69K
W108Y
Y72A
D81K
W96A
α-RhlR

Figure S3

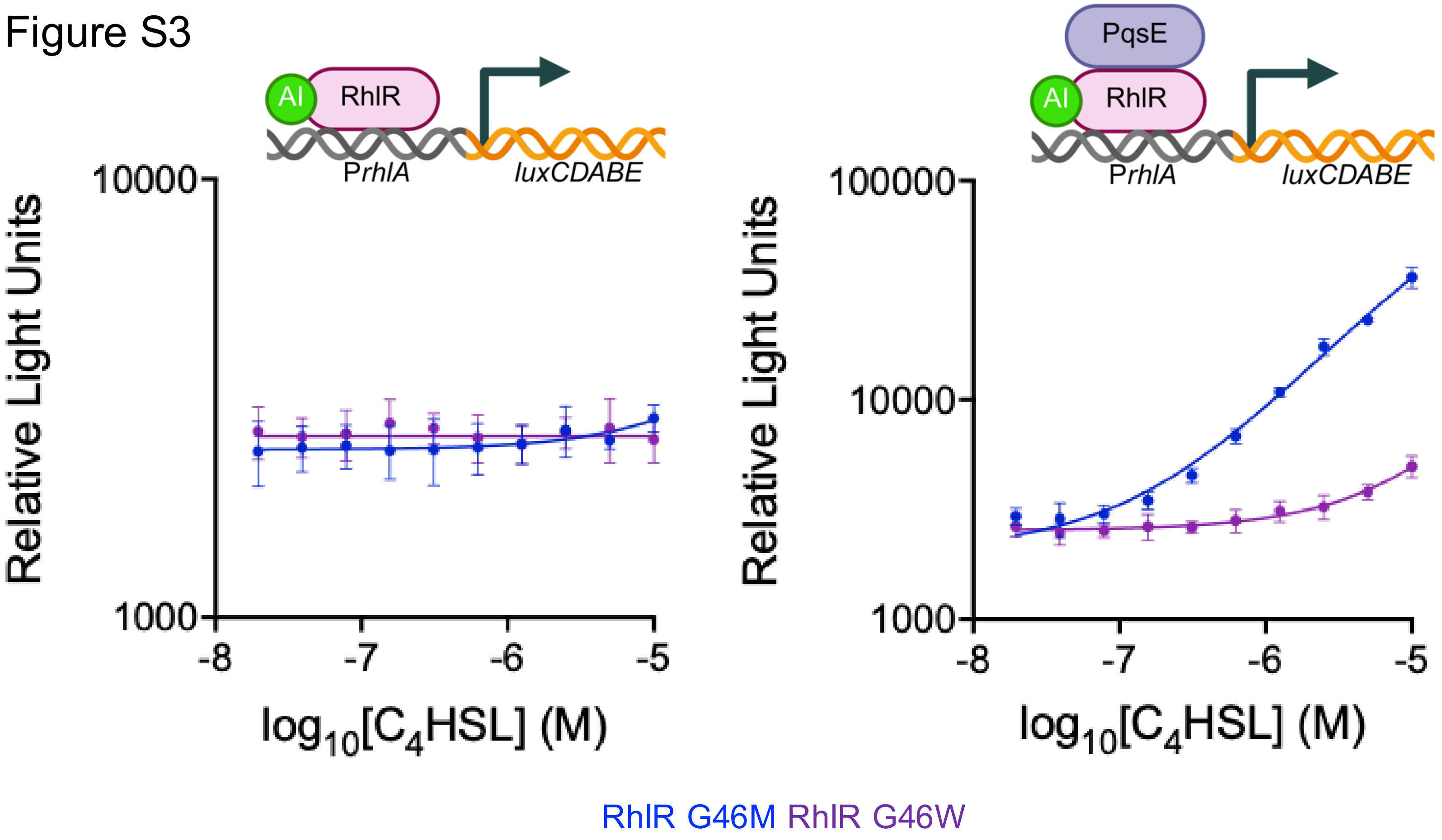

AI
RhlR
PrhlA
luxCDABE
10000
1000
Relative Light Units
-8
-7
-6
-5
log10[C4HSL] (M)
PqsE
AI
RhlR
PrhlA
luxCDABE
100000
10000
1000
Relative Light Units
-8
-7
-6
-5
log10[C4HSL] (M)
RhlR G46M RhlR G46W

Figure S4

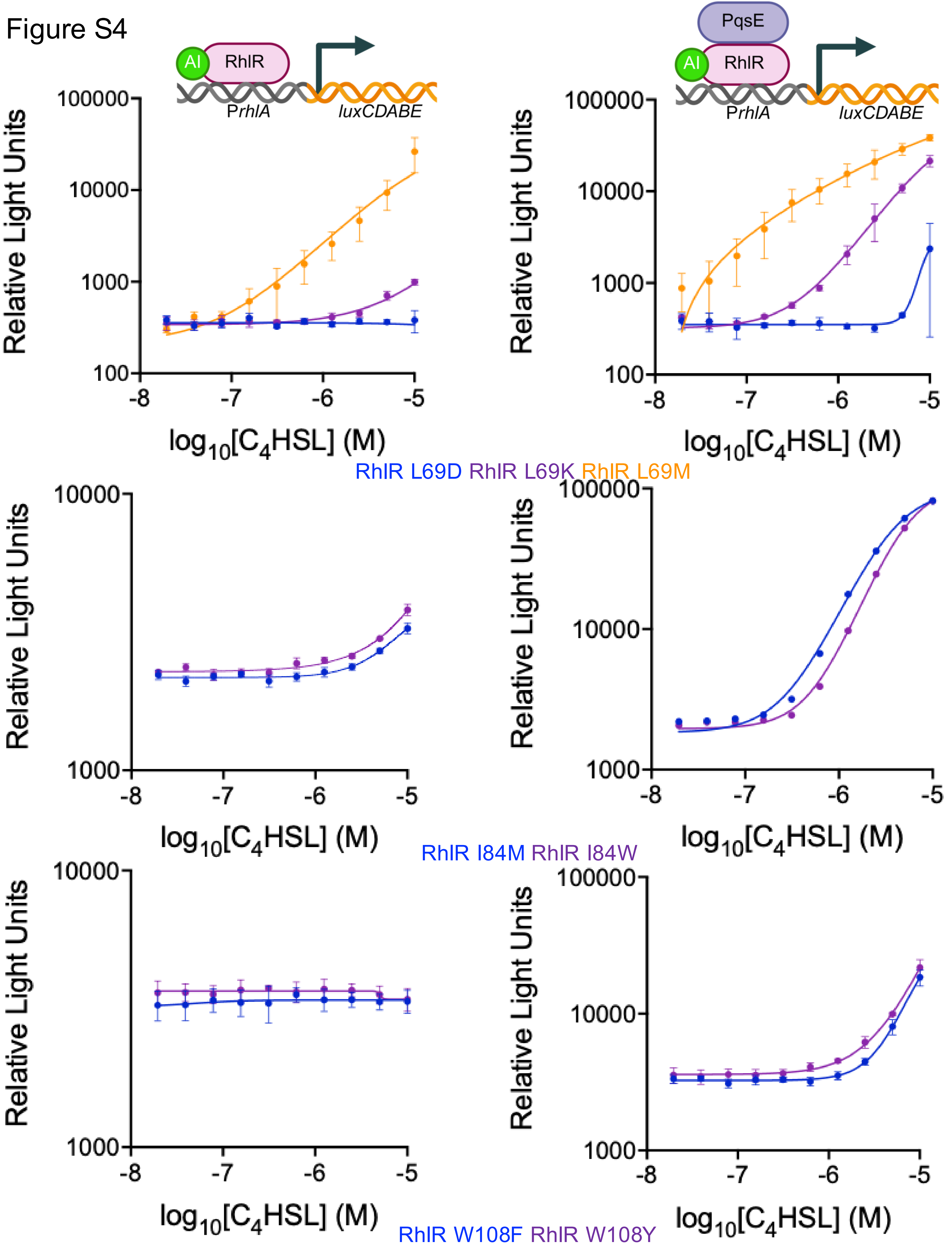

AI
RhlR
PrhlA
luxCDABE
PqsE
Relative Light Units
log10[C4HSL] (M)
RhlR L69D RhlR L69K RhlR L69M
RhlR I84M RhlR I84W
RhlR W108F RhlR W108Y

Figure S5

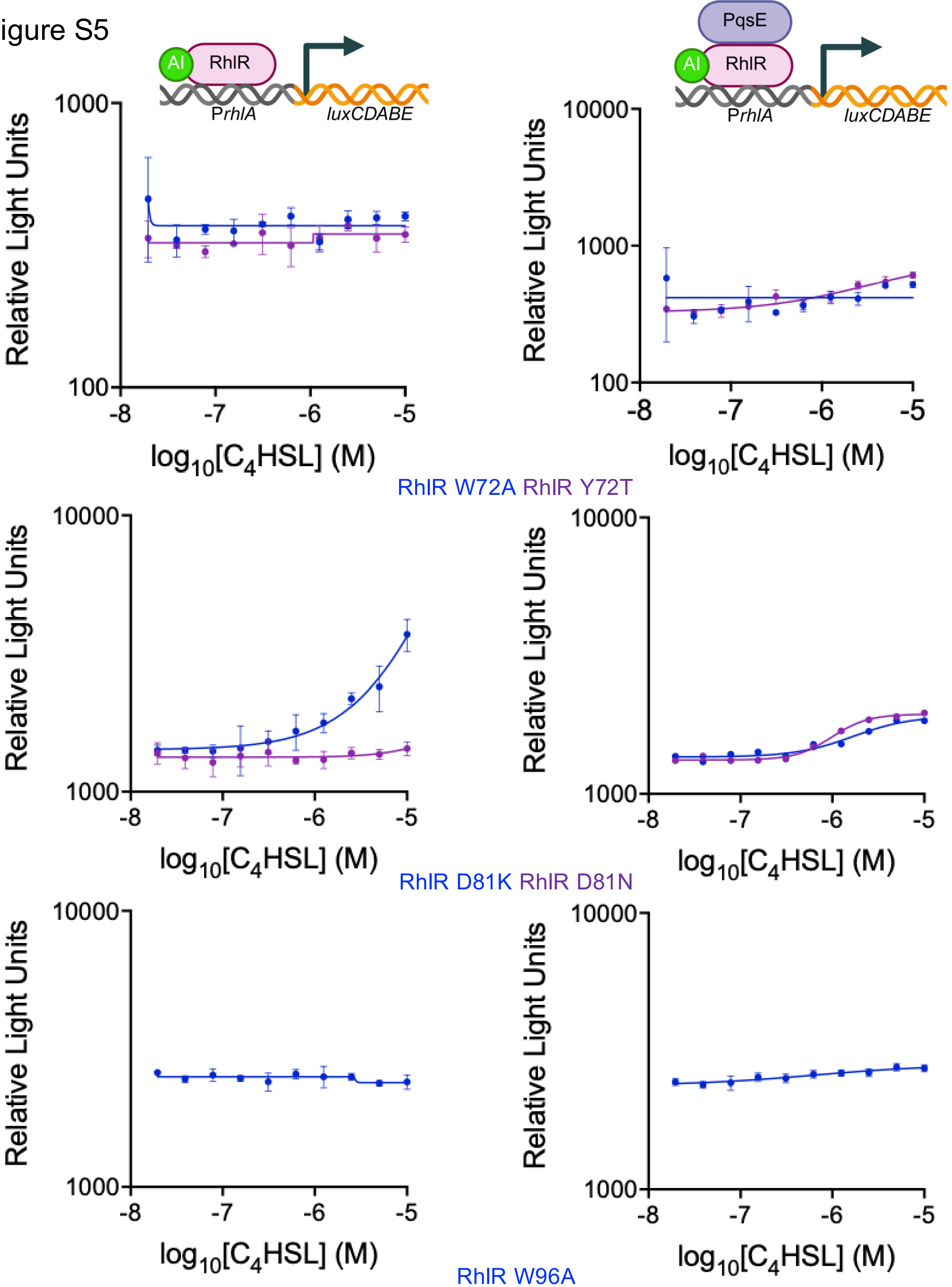

PqsE
AI
RhlR
PrhlA
luxCDABE
Relative Light Units
log10[C4HSL] (M)
RhlR W72A RhlR Y72T
RhlR D81K RhlR D81N
RhlR W96A

Figure S6

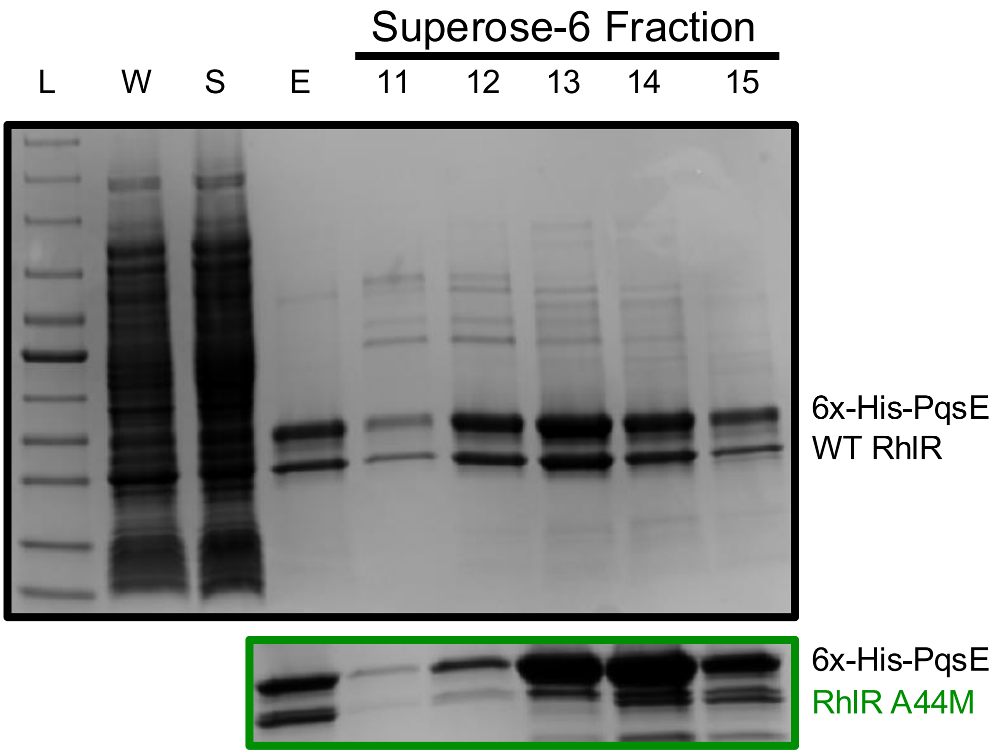
Superose-6 Fraction
L
W
S
E
11
12
13
14
15
6x-His-PqsE
WT RhlR
6x-His-PqsE
RhlR A44M

Figure S7

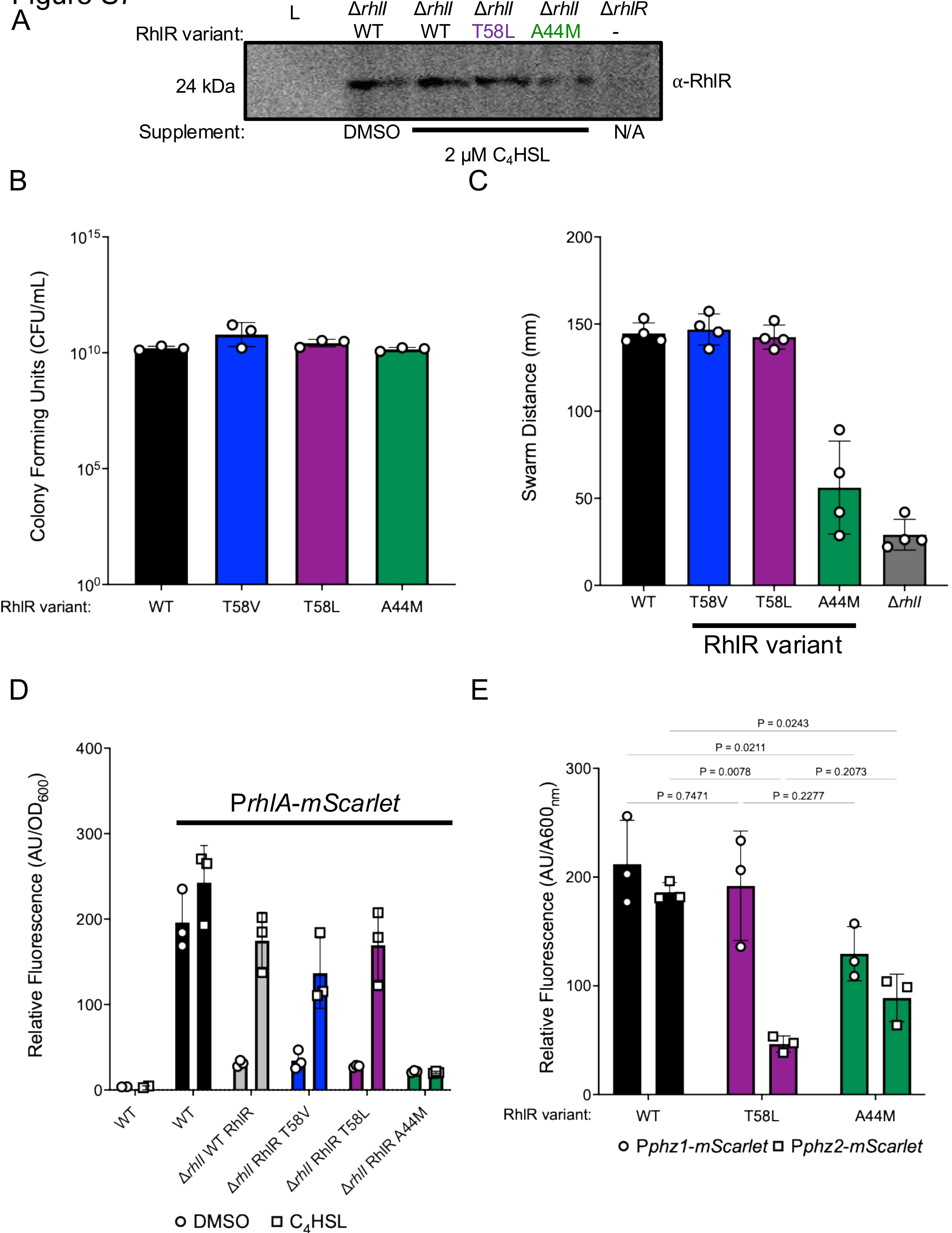
A
L
ΔrhlI WT
ΔrhlI WT
ΔrhlI T58L
ΔrhlI A44M
ΔrhlR -
RhlR variant:
24 kDa
α-RhlR
Supplement:
DMSO
2 μM C4HSL
N/A
B
Colony Forming Units (CFU/mL)
RhlR variant:
WT
T58V
T58L
A44M
C
Swarm Distance (mm)
WT
T58V
T58L
A44M
ΔrhlI
RhlR variant
D
Relative Fluorescence (AU/OD600)
PrhlA-mScarlet
WT
WT
ΔrhlI WT RhlR
ΔrhlI RhlR T58V
ΔrhlI RhlR T58L
ΔrhlI RhlR A44M
DMSO
C4HSL
E
Relative Fluorescence (AU/A600nm)
P = 0.0243
P = 0.0211
P = 0.0078
P = 0.2073
P = 0.7471
P = 0.2277
RhlR variant:
WT
T58L
A44M
Pphz1-mScarlet
Pphz2-mScarlet

Figure S8

A

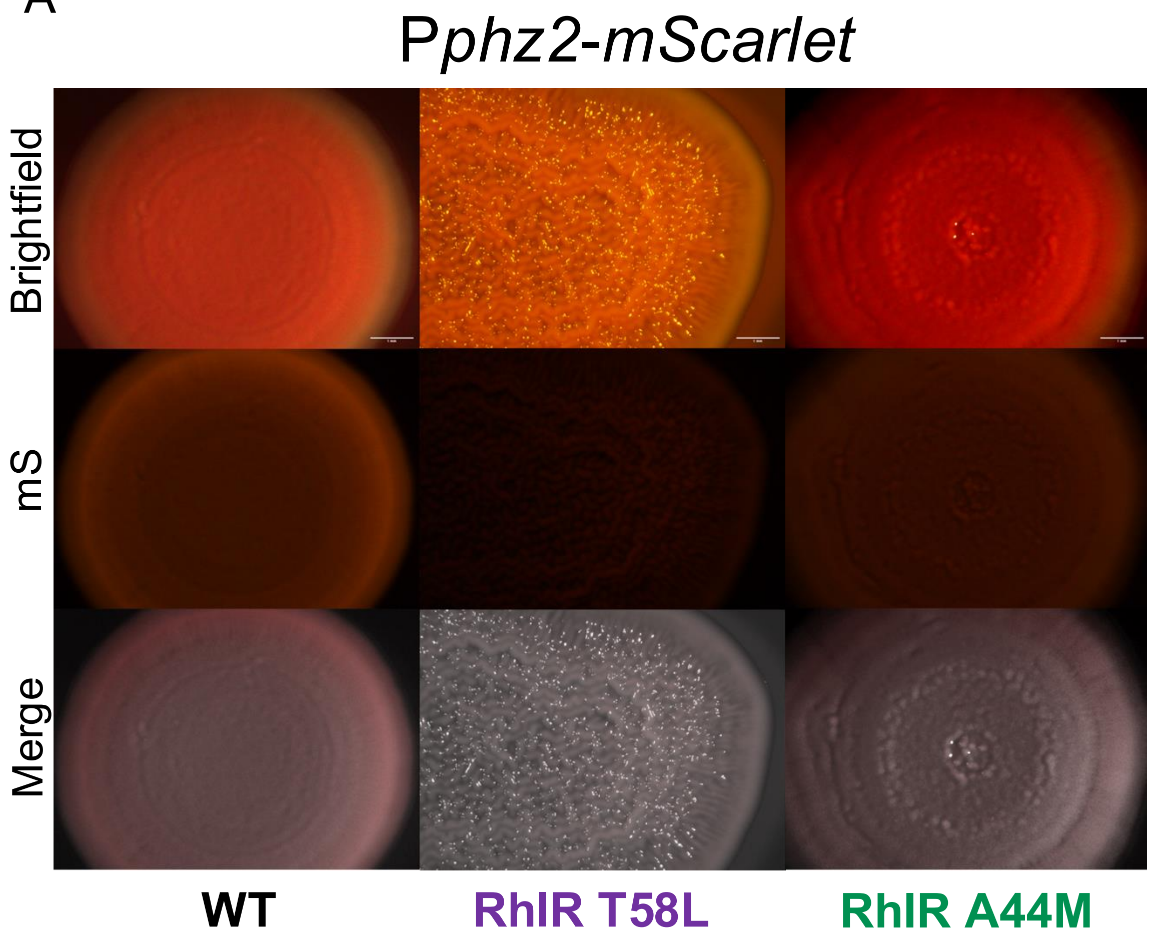

Pphz2-mScarlet
Brightfield
mS
Merge
WT
RhlR T58L
RhlR A44M

B

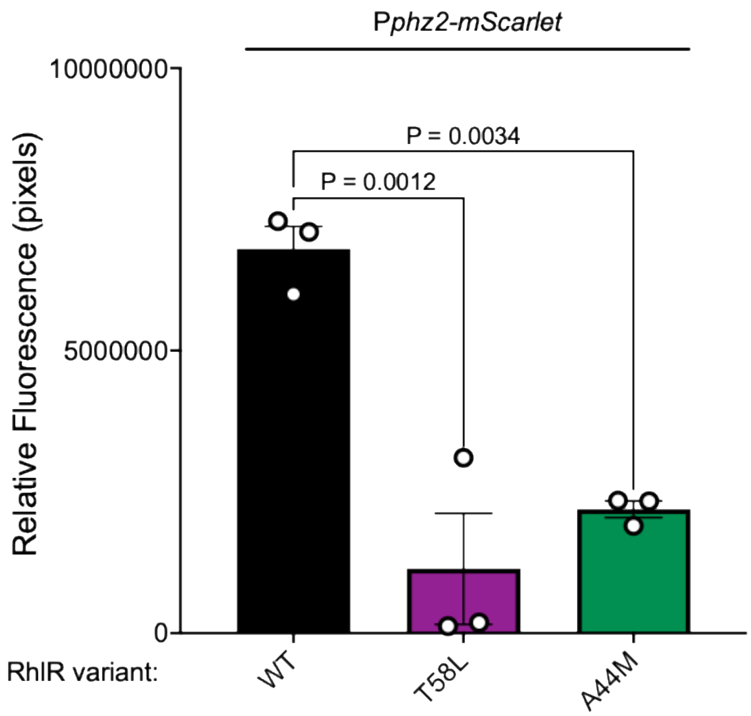

Pphz2-mScarlet
10000000
5000000
0
Relative Fluorescence (pixels)
P = 0.0034
P = 0.0012
RhlR variant:
WT
T58L
A44M

Figure S9

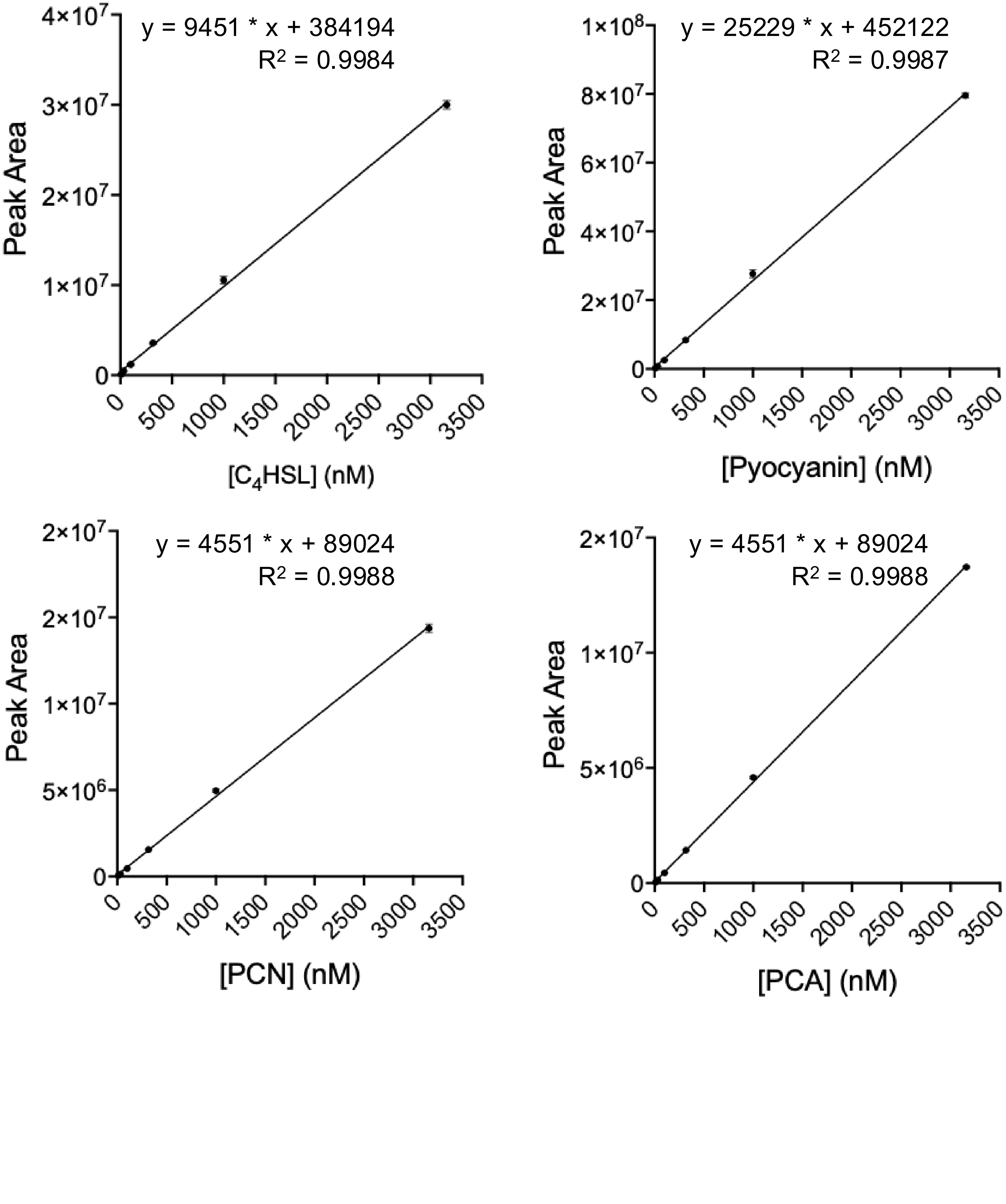
y = 9451 * x + 384194
R2 = 0.9984
Peak Area
[C4HSL] (nM)
y = 25229 * x + 452122
R2 = 0.9987
Peak Area
[Pyocyanin] (nM)
y = 4551 * x + 89024
R2 = 0.9988
Peak Area
[PCN] (nM)
y = 4551 * x + 89024
R2 = 0.9988
Peak Area
[PCA] (nM)

Figure S10

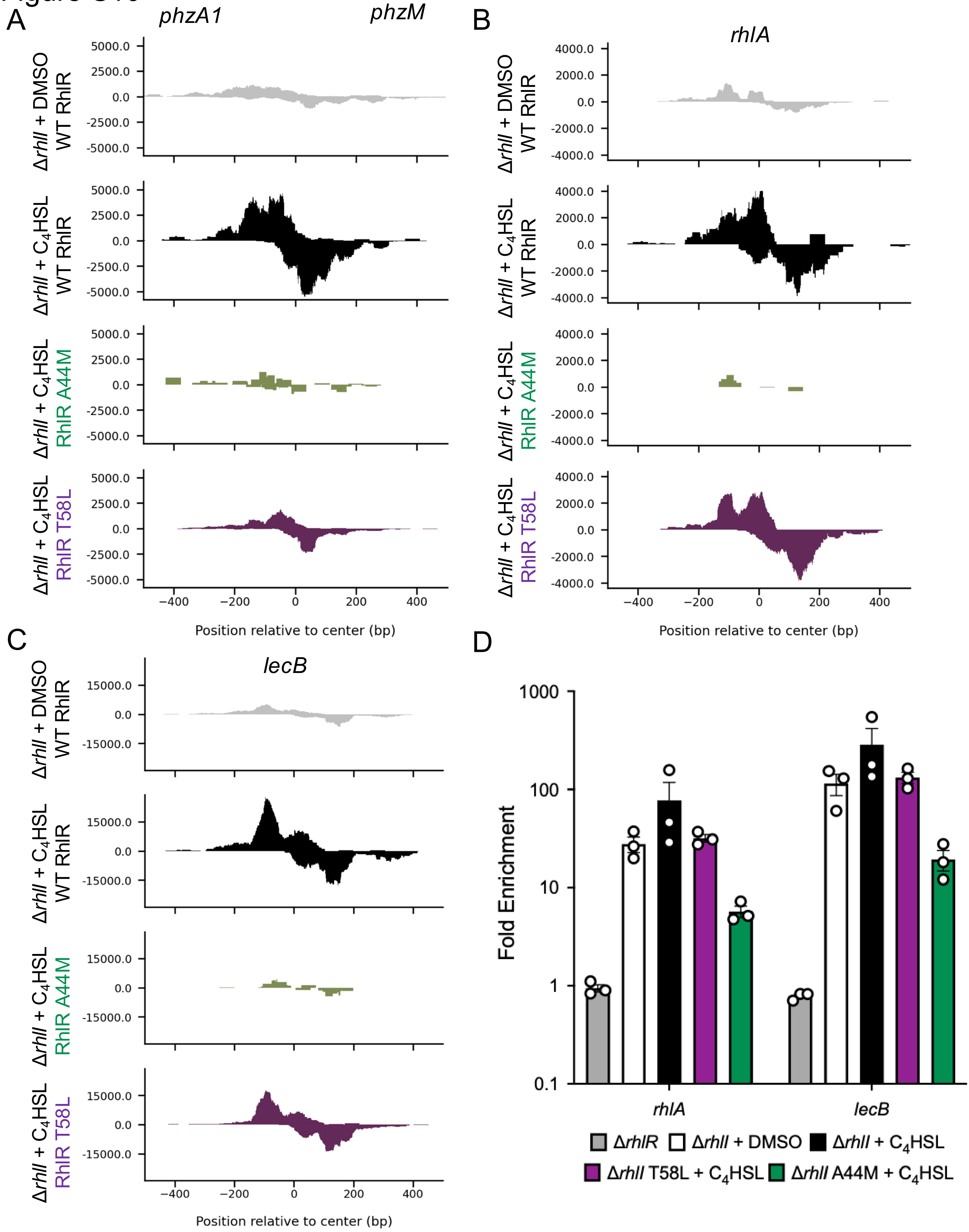
A
phzA1
phzM
B
rhlA
C
lecB
D
ΔrhlI + DMSO WT RhlR
ΔrhlI + C4HSL WT RhlR
ΔrhlI + C4HSL RhlR A44M
ΔrhlI + C4HSL RhlR T58L
Position relative to center (bp)
Fold Enrichment
rhlA
lecB
ΔrhlR
ΔrhlI + DMSO
ΔrhlI + C4HSL
ΔrhlI T58L + C4HSL
ΔrhlI A44M + C4HSL
