# Supplementary material for: RhlR quorum-sensing receptor ligand sensitivity regulates the differential expression of phenazine genes in Pseudomonas aeruginosa
Source: J Bacteriol. 2026 Apr 8;208(5):e00013-26. doi: 10.1128/jb.00013-26 (PMC13192270; doi:10.1128/jb.00013-26)
Supplement: Supplemental legends — Legends for Figures S1 to S10. [file jb.00013-26-s0002.docx]

Title: RhlR quorum-sensing receptor ligand sensitivity regulates the differential expression of phenazine genes in *Pseudomonas aeruginosa*

Autumn N. Pope^a^, Varun R. Bavda^a^, Megan L. Schumacher^b^, Alicia G. Mendoza^a,b^, Anne M. Stringer^a^, Caleb P. Mallery^b^, Anna Czachor^a,d^, Amanda F. Kurtz^a^, Biqing Liang^b^, Xia Ke^c^, Joseph T. Wade^a,b,d^, and Jon E. Paczkowski^a,b,d,#^

^a^Division of Genetics, Wadsworth Center, New York State Department of Health, Albany, NY, USA

^b^Department of Biomedical Sciences, University at Albany, College of Integrated Health Sciences, Albany, NY, USA

^c^Center for Biotechnology and Interdisciplinary Studies, Rensselaer Polytechnic Institute, Troy, NY, USA

^d^The RNA Institute, University at Albany, College of Arts and Sciences, Albany, New York, USA

**SUPPORTING INFORMATION**

**Table S1.** Read counts for all mRNA from RNA-seq experiments for all strains.

**Table S2.** Strains and plasmids used in this study.

**Table S3.** Oligonucleotides used in this study.

**Figure S1. Structural analysis of the RhlR LBP.** Zoom in view of the RhlR (pink) LBP. C_6_HSL (salmon), which was computationally docked into the WT RhlR LBP (PDB: 8DQ0) (top) and mBTL (green) from experimental results (PDB: 8DQ0) are shown. Residues A44, G46, T58, Y64, W68, L69, Y72, D81, I84, W96, W108, and V133 are shown as sticks. Residues are colored based on variant responses to C_4_HSL: orange = substitutions are recalcitrant to C_4_HSL; magenta = substitutions are hypersensitive to C_4_HSL; gray = substitutions are hyposensitive to C_4_HSL; dark green = phenylalanine substitutions were previously described and lead to a “constitutive” state for RhlR.

**Figure S2. Expression of RhlR variants in the *E. coli* reporter assay.** WT RhlR and RhlR variants expressed from the pBAD-A vector in *E. coli* as measured by western blot analysis using a polyclonal antibody for RhlR. An empty vector (EV) control was used to assess antibody specificity.

**Figure S3. RhlR G46 variants are less responsive to C_4_HSL than WT RhlR.** RhlR-controlled bioluminescence was measured in *E. coli*. Arabinose-inducible RhlR was expressed from one plasmid and a p*rhlA*-*luxCDABE* reporter construct was carried on a second plasmid to monitor transcriptional activity. 0.1% arabinose was used to induce RhlR. RhlR-dependent bioluminescence was measured for WT RhlR (black) and RhlR variants (G46M = blue, G46W = purple) in response to increasing concentrations (µM) of C_4_HSL with or without PqsE.

**Figure S4. RhlR L69/I84/W108 variants are less responsive to C_4_HSL than WT RhlR.** RhlR-controlled bioluminescence was measured in *E. coli*. Arabinose-inducible RhlR was expressed from one plasmid and a p*rhlA*-*luxCDABE* reporter construct was carried on a second plasmid to monitor transcriptional activity. 0.1% arabinose was used to induce RhlR. RhlR-dependent bioluminescence was measured for WT RhlR (black) and RhlR variants**; top)** (L69D = blue, L69K = purple, L69M = orange), **middle)** (I84M = blue, I84W = purple), and **bottom)** (W108F = blue, W108Y = purple) in response to increasing concentrations (µM) of C_4_HSL with or without PqsE.

**Figure S5. RhlR Y72/D81/W96 variants are non-responsive to C_4_HSL than WT RhlR.** RhlR-controlled bioluminescence was measured in *E. coli*. Arabinose-inducible RhlR was expressed from one plasmid and a p*rhlA*-*luxCDABE* reporter construct was carried on a second plasmid to monitor transcriptional activity. 0.1% arabinose was used to induce RhlR. RhlR-dependent bioluminescence was measured for WT RhlR (black) and RhlR variants; **top)** (Y72A = blue, Y72T = purple), **middle)** (D81K = blue, D81N = purple), and **bottom)** (W96A = blue) in response to increasing concentrations (µM) of C_4_HSL with or without PqsE.

**Figure S6. Purification of WT RhlR:C_6_HSL-PqsE and RhlR A44M-PqsE.** Representative SDS-PAGE gel of whole-cell lysate (W), supernatant (S), and elution (E) for Ni-NTA purification of WT RhlR:C_6_HSL-PqsE as well as fractions 11-15 (Figure 3C) from Superose-6 size-exclusion chromatography for RhlR:C6HSL-PqsE and RhlR A44M-PqsE.

**Figure S7. RhlR T58V/L and A44M variants are expressed and differentially regulate *rhlA* and swarming. A)** Expression of WT RhlR and RhlR variants from its native locus in *P. aeruginosa* as measured by western blot analysis using a polyclonal antibody for RhlR. **B)** Colony forming units of cells collected from colony biofilms for pyocyanin extraction shown in Figure 5C, 4D. **C)** Branch length measurements of strains expressing WT RhlR and RhlR variants compared to Δ*rhlI* grown on swarming media. Branch length was measured using ImageJ by establishing the center of the initial inoculant and measuring in a direct line to the tip of the branch. **D)** Expression of plasmid-borne a p*rhlA*-mSacrlet reporter in WT RhlR and RhlR variants compared to Δ*rhlI*. Statistical analyses for all assays were performed using an ordinary one-way ANOVA with a Tukey’s multiple comparisons test; comparisons that were deemed not significant by these analyses are not shown. **E)** WT *P. aeruginosa* strains expressing WT RhlR (black), RhlR T58L (purple) and RhlR A44M (green) with the *phz1* (circle) and *phz2* (square) promoters fused to mScarlet integrated at the *attB* locus. Statistical analyses for all assays were performed using an ordinary one-way ANOVA with a Tukey’s multiple comparisons test. Comparisons that were deemed not significant by these analyses are not shown.

**Figure S8. Differential phenazine expression in biofilms expressing RhlR variants. A)** Representative brightfield and fluorescent images of Congo red colony biofilm plates for strains of *P. aeruginosa* expressing WT RhlR, RhlR T58L, and RhlR A44M with the *phz2* promoter fused to *mScarlet* integrated at the *attB* locus. Red signal indicates the expression of *phz2*-*mScarlet*. Images were merged using ImageJ. **B)** Quantification of fluorescent signal from three independent replicates from **A)** using a non-fluorescent strain of PA14 as a control. Statistical analyses for all assays were performed using an ordinary one-way ANOVA with a Tukey’s multiple comparisons test. Comparisons that were deemed not significant by these analyses are not shown.

**Figure S9. Standards and controls for LC-MS experiments.** Calibration standards for C_4_HSL, pyocyanin, phenazine-1-carboxylic acid (PCA), and phenazine-1-carboxamide (PCN) performed at 10, 31.6, 100, 316, 1000, 3160 nM. The area of the peak was measured using Xcalibur and plotted as a function of molecule concentration. A simple linear aggression analysis was calculated for each curve to determine the slope, which was used to calculate the values determined in Figure 5C.

**Figure S10. RhlR variant promoter occupancy.** Occupancy plot profiles showing the level of mapped ChIP-seq reads +/- 500 bp of the called peak centers for the designated strains at the **A)** *phzA1* and *phzM*, **B)** *rhlA*  and **C)** *lecB* promoters. **D)** ChIP-qPCR analysis of WT RhlR and RhlR variant promoter binding to the regions upstream of *lecB* and *rhlA* to measure the relative levels of RhlR binding to DNA sites in a Δ*rhlI* strain containing 2 μM C_4_HSL. All data were normalized to a non-specific DNA control site.
